# DiffINR: conditional neural field diffusion for deformation-driven volumetric image estimation from limited measurements

**DOI:** 10.1088/1361-6560/aeaaa0

**Published:** 2026-10-09

**Authors:** Xiaoxue Qian, Hua-Chieh Shao, You Zhang

**Affiliations:** The Medical Artificial Intelligence and Automation (Maia) Laboratory, Department of Radiation Oncology, University of Texas Southwestern Medical Center, Dallas, TX, 75390, United States of America

**Keywords:** Implicit neural representation (INR), diffusion model, volumetric image estimation, limited-data imaging, patient-specific adaptation

## Abstract

*Objective.* We propose DiffINR for rapid deformation-driven estimation of patient-specific target volumes from limited measurements. DiffINR uses a coordinate-based implicit neural representation (INR) to estimate a dense deformation vector field (DVF) that warps a high-quality prior volume (source) to the target anatomy, with patient-specific INR initialization provided by a population-based conditional hyperdiffusion model. *Approach.* During offline training, INRs were optimized on fully sampled source-target image volume pairs to represent DVFs. A Transformer diffusion model, hyperdiffusion, learned the distribution of the resulting INR weights. The model was jointly conditioned on anatomical features extracted from the training source and target volumes by a foundation model and on features encoded directly from limited measurements. At inference, hyperdiffusion generated INR initial weights, which were further fine-tuned by patient-specific optimization using measurement-domain fidelity and DVF-smoothness losses. We evaluated DiffINR on two tasks: cone-beam computed tomography (CBCT) estimation from limited-angle x-ray projections and real-time volumetric magnetic resonance imaging (MRI) estimation from ultra-sparse k-space data. *Main Results.* For orthogonal-view 90° CBCT estimation, DiffINR achieved a structural similarity index measure (SSIM) of 0.962 ± 0.021, relative error of 6.84% ± 1.70%, and target registration error of 3.61 ± 1.27 mm, outperforming the iterative methods, population-based models, and randomly initialized INR baselines. For volumetric MRI estimation with 13 radial spokes per slice, DiffINR achieved an SSIM of 0.985 ± 0.007, Dice coefficient of 0.89 ± 0.04, and center-of-mass error of 1.30 ± 1.09 mm. DiffINR showed high robustness to data distribution shifts at test time, including dataset changes, limited-angle setting variations, and different MRI sampling sparsity levels. Test-time runtime was 23 s for CBCT estimation and 27 s for MRI estimation, compared with 795 s and 928 s, respectively, for INR optimization from scratch. *Significance.* By combining hyperdiffusion-based INR initialization with patient-specific data consistency, DiffINR achieves fast and accurate deformation-driven volumetric image estimation.

## 1. Introduction

Volumetric imaging, which provides comprehensive three-dimensional (3D) representations of patient anatomy, has become indispensable in medical diagnosis, treatment planning, and image-guided radiotherapy (Dawson and Jaffray 2007, Verellen *et al* 2008, Jaffray 2012). In time-sensitive clinical scenarios, rapid and accurate volumetric imaging is particularly important for applications such as magnetic resonance (MR)-guided radiotherapy (Pollard *et al* 2017), cone-beam computed tomography (CBCT)-guided target localization and treatment verification (Srinivasan *et al* 2014), motion monitoring (Bertholet *et al* 2019), and adaptive treatment delivery (Yan *et al* 1997). To satisfy clinical workflow constraints, data acquisition is often accelerated, resulting in limited measurements, including undersampled k-space data in MR imaging to shorten scan time and increase temporal resolution (Lustig *et al* 2008), and limited-angle projections in CBCT to reduce imaging time and dose and address acquisition geometry constraints (Xie *et al* 2024). Although these strategies enhance clinical applicability, the limited measurements provide insufficient information for high-fidelity volumetric reconstruction, which may lead to image artifacts, anatomical uncertainty, and compromised target localization (Hatamikia *et al* 2022, Tavakkoli and Noseworthy 2025). Consequently, accurate and reliable volumetric imaging under limited measurement conditions remains a fundamental challenge in time-sensitive image-guided radiotherapy and adaptive treatment workflows.

Conventional methods address volumetric reconstruction from limited measurements by incorporating explicit priors or physical imaging constraints to regularize the inherently ill-posed inverse problem, including compressed sensing (Lustig *et al* 2008), low-rank modeling (Otazo *et al* 2015), motion-compensated reconstruction (Lingala *et al* 2014), and deformation-driven estimation (Huang *et al* 2018). Compressed sensing and low-rank modeling methods improve reconstruction robustness by enforcing sparsity or spatiotemporal redundancy (Graff and Sidky 2015, Ye 2019). However, these predefined assumptions may over-simplify image representations and introduce over-smoothness in the reconstruction of complex anatomical structures. Motion-compensated reconstruction and deformation-driven estimation further exploit temporal correspondence or high-quality prior images to preserve anatomical fidelity, making them particularly useful for dynamic and longitudinal imaging tasks (Sotiras *et al* 2013). Despite these advantages, performance depends heavily on accurate motion estimation and deformation modeling. In addition, conventional model-based approaches often rely on time-consuming iterative optimization, which may limit their ability to achieve efficient online deployment.

Recent advances in deep learning have enabled data-driven volumetric image reconstruction and estimation methods to learn more expressive representations while reducing reliance on the iterative optimization used in conventional model-based approaches. These methods typically learn mappings from sparse or incomplete measurements to volumetric representations using population-level training datasets, enabling fast inference after training. Existing methods generally fall into two categories: reconstruction-based methods (Hammernik *et al* 2018, Lu *et al* 2021), which directly reconstruct target volumes from acquired measurements; and deformation-driven methods (Balakrishnan *et al* 2019, Zhang 2021), which estimate motion fields from observations to deform prior images into target volumes. Deformation-driven methods are well suited for radiation oncology applications, where the existence of high-quality prior images (e.g., simulation CT/MR images) naturally supports anatomically faithful volumetric estimation, motion tracking, and target localization. VoxelMorph (Balakrishnan *et al* 2019) is a widely used learning-based registration framework that directly predicts dense deformation vector fields (DVFs) from image pairs, enabling substantially faster inference than conventional iterative registration methods. Building on this learning-based registration paradigm, the 2D3D-RegNet framework (Zhang 2021) was proposed to estimate volumetric CBCTs by learning DVFs to deform prior images based on limited-angle x-ray projections. KS-RegNet (Shao *et al* 2022) is a k-space-driven, learning-based registration framework for real-time volumetric MRI motion estimation. These population-based methods, which are trained using a cohort of patient cases, can achieve rapid, sub-second inference for real-time volumetric image estimation. However, their accuracy depends on the representativeness of training data, which may limit their generalization to unseen anatomical variations, imaging protocols, or patient-specific characteristics at test time.

Implicit neural representations (INRs) (Sitzmann *et al* 2020), typically parameterized by a multilayer perceptron (MLP) that maps spatial and/or temporal coordinates to signals, provide compact and continuous representations for objects including volumetric medical images, DVFs, and radiation dose distributions. Instead of learning a fixed cohort-level mapping, INRs optimize lightweight neural network parameters directly for each new case (‘one-shot’ learning), enabling test-time subject-specific modeling of anatomy and motion (Byra *et al* 2023, Han *et al* 2023, Van Harten *et al* 2023). Thus, distinct from population-driven deep learning models, INR-based methods are well suited to address the generalizability challenges. One of the early INR-based frameworks for deformable medical image registration used an MLP to represent the dense DVF between a pair of images (Wolterink *et al* 2022). STINR-MR (Shao *et al* 2024a) and PMF-STINR (Shao *et al* 2024b) applied patient-specific spatiotemporal INR learning to time-resolved MR and CBCT reconstruction, respectively. DREME (Shao *et al* 2025a) and DREME-MR (Shao *et al* 2025b) further integrated patient-specific INR reconstruction with convolutional neural network-based motion encoding for real-time volumetric image reconstruction and motion tracking. However, optimizing INRs from scratch remains computationally demanding and can be sensitive to parameter initialization under sparse or noisy observations (Tancik *et al* 2021). To improve the optimization efficiency and stability, HB-2D3DReg (Qian *et al* 2025) and cMeta-INR (Qian *et al* 2026) incorporated population-level priors into patient-specific INR optimization. In Hb-2D3DReg, a population-based 2D-3D registration network was used to provide a coarse initialization of the DVF for limited-angle CBCT estimation, thereby accelerating the subsequent INR refinement. In cMeta-INR, cohort-informed meta-learning was introduced to learn transferable INR templates for rapid adaptation to undersampled MR k-space data, enabling deformation-driven real-time volumetric MRI estimation. These studies demonstrated that integrating cohort knowledge with patient-specific INR optimization accelerated test-time convergence speed and increased the accuracy of deformation-driven volumetric image estimation.

Despite their advances, existing INR acceleration strategies are often tailored to specific imaging settings, which may limit their flexibility across different modalities and measurement conditions. Diffusion models (Ho *et al* 2020) offer a promising alternative by learning the complex distribution of INR parameters, enabling robust and efficient initialization for subsequent optimization. A generative diffusion model was proposed for the unconditional synthesis of implicit neural fields (ERKOç *et al* 2023). Neural network diffusion generates high-performing neural network parameters through latent parameter diffusion (Wang *et al* 2024). NeRV-Diffusion (Ren *et al* 2025) extends diffusion-based INR parameter generation to video synthesis. However, these approaches mainly focus on unconditional parameter generation without conditioning on patient-specific observations or measurements. Unlike conventional generative tasks that focus on novel sample synthesis, limited-sampled volumetric image estimation requires rapid adaptation to a specific patient under scarce measurements. Motivated by these considerations, we propose DiffINR, a conditional hyperdiffusion framework for patient-specific, deformation-driven volumetric image estimation from limited measurements. DiffINR operates in the parameter space of implicit neural fields and predicts case-specific INR initialization parameters for subsequent test-time refinement. Specifically, a collection of INRs is first optimized offline based on fully-sampled training volumetric images to capture population-level deformation patterns. Based on the resulting INR parameters, a conditional hyperdiffusion model is then trained with patient-specific limited observations. During inference, the diffusion process predicts patient-specific INR parameters by conditioning on two complementary sources of information: measurement-aware features encoded from patient-specific limited observations, and foundation model-derived semantic priors extracted from the source/target image pairs. By guiding diffusion in INR weight space with these fused conditions, DiffINR provides an informed and robust initialization for efficient test-time adaptation. In this way, DiffINR provides a flexible framework that combines population-informed INR initialization with patient-specific test-time optimization, without requiring the overall deformation-driven image estimation workflow to be redesigned for different measurement configurations or data-limited scenarios. For a new application or measurement setting, the same overall framework can be retained, while the hyperdiffusion model generally needs to be retrained on data representative of that specific scenario.

DiffINR is designed as a framework for measurement-limited, deformation-driven volumetric image estimation under specific training- and test-data availability conditions. It is applicable to imaging tasks in which representative fully sampled source-target volume pairs are available for offline INR parameter-space construction and hyperdiffusion training, while a fully sampled source volume and limited target measurements are available for a new case at test time. The framework is not intrinsically restricted to a specific imaging modality like CBCT or MRI. For a new imaging modality, the same training-and-inference workflow can be followed using the corresponding fully sampled training volumes and limited test-time measurements. The main contributions of this study are summarized as follows.

1) We introduce a conditional patient-specific adaptation approach for limited-data volumetric imaging by performing conditional generative modeling directly in the INR parameter space.
2) DiffINR integrates two complementary conditioning features: image-domain semantic priors extracted from source/target image pairs and measurement-aware features encoded from limited observations, enabling fast INR parameter initialization. By using diffusion-predicted INR parameters to initialize subsequent patient-specific refinement, DiffINR effectively combines population-level priors with individualized adaptation, improving convergence efficiency, robustness, and anatomical fidelity under severe data limitations.
3) We demonstrate the general applicability of DiffINR in two representative imaging tasks: limited-angle lung CBCT estimation and undersampled cardiac cine MRI estimation, which involve different imaging modalities, measurement domains, acquisition processes, and motion patterns.

## 2. Materials and methods

### 2.1. Problem formulation

Let ${\chi _{{\mathrm{tg}}}}$ denote the limited measurements acquired for a patient, such as undersampled MR k-space data or limited-angle CBCT projections. Let ${v_{{\mathrm{sc}}}} \in {\mathcal{R}^{H \times W \times D}}$ denote an available, high-quality prior image serving as the source image, and ${v_{{\mathrm{tg}}}} \in {\mathcal{R}^{H \times W \times D}}$ denote the target volumetric image to be estimated. The goal of patient-specific volumetric image estimation is to recover the target image ${v_{{\mathrm{tg}}}}$ from the source image ${v_{{\mathrm{sc}}}}$ by leveraging the limited target measurements ${\chi _{tg}}$. Rather than directly modeling voxel intensities, we formulate the problem as a deformable registration-based image estimation. A coordinate-based INR, parameterized by ${\boldsymbol{\theta }}$, is used to predict a dense DVF ${\boldsymbol{d}_{\boldsymbol{\theta }}}\left( {\boldsymbol{r}} \right)$, where ${\boldsymbol{d}} \in {\mathcal{R}^3}$ denotes voxel-wise movement defined on a spatial coordinate r. The predicted DVF is then applied to deform the source image through a spatial transformation ${\text{ }}\mathcal{T}\left( \cdot \right)$, yielding the estimated target image: ${\hat v_{{\mathrm{tg}}}} = \mathcal{T}\left( {{v_{{\mathrm{sc}}}},{d_\theta }} \right)$. Accordingly, the deformation-driven target image estimation can be formulated as:

\begin{equation*}{\theta ^*} = {\mathrm{arg}}\mathop {\min }\limits_\theta {\mathcal{L}_{{\mathrm{data}}}}\left( {{{\hat \chi }_{{\mathrm{tg}}}},{ }{\chi _{{\mathrm{tg}}}}} \right) + {\lambda _1}{\mathcal{L}_{{\mathrm{reg}}}}\left( {{d_\theta }} \right),\end{equation*}

where ${\mathcal{L}_{{\mathrm{data}}}}$ denotes the data fidelity loss between the predicted measurements ${\hat \chi _{{\mathrm{tg}}}} = \mathcal{A}\left( {{{\hat v}_{{\mathrm{tg}}}}} \right)$ and the acquired measurements ${\chi _{tg}}$, and $\mathcal{A}\left( \cdot \right)$ is the measurement operator. ${\mathcal{L}_{{\mathrm{reg}}}}$ is a regularization term imposed on the DVF to encourage spatial smoothness, and ${\lambda _1}$ controls the trade-off between the data fidelity and the DVF regularization.

Although INR can provide compact and continuous representations of dense motion fields, optimizing ${\boldsymbol{\theta }}$ from random initialization for each patient is time-consuming, while INR optimization can also be sensitive to parameter initialization (Sitzmann *et al* 2020, Tancik *et al* 2021), particularly when constrained by limited measurements. To address this challenge, DiffINR was developed to learn the conditional distribution of optimized INR parameters through a diffusion model and use the predicted weights as informative initialization for efficient patient-specific refinement.

### 2.2. Overview of DiffINR

The overall DiffINR framework, as illustrated in figure 1, consists of three stages: offline INR parameter space construction, conditional hyperdiffusion learning, and test-time patient-specific refinement. First, in stage a, an INR is optimized for each source-target pair of fully sampled volumetric images from the training cohort to capture the corresponding inter-image DVF. This process produces a collection of optimized INR parameters that characterize population-level deformation patterns (figure 1(a)). Second, in stage b, a conditional hyperdiffusion model is trained in the INR parameter space constructed in stage a using the same training cohort. The model learns the distribution of optimized INR parameters conditioned on foundation model-derived semantic features from the source-target image pairs and measurement-aware features extracted from limited observations (figure 1(b)). Finally, in stage c, the conditional hyperdiffusion model trained in stage b predicts the INR weights for a new test case based on the available measurements. These predicted weights initialize the INR for rapid patient-specific refinement under the data-fidelity objective (figure 1(c)). This design allows DiffINR to combine population-level deformation priors with patient-specific observations for accurate and efficient volumetric image estimation from limited measurements.

**Figure 1.**
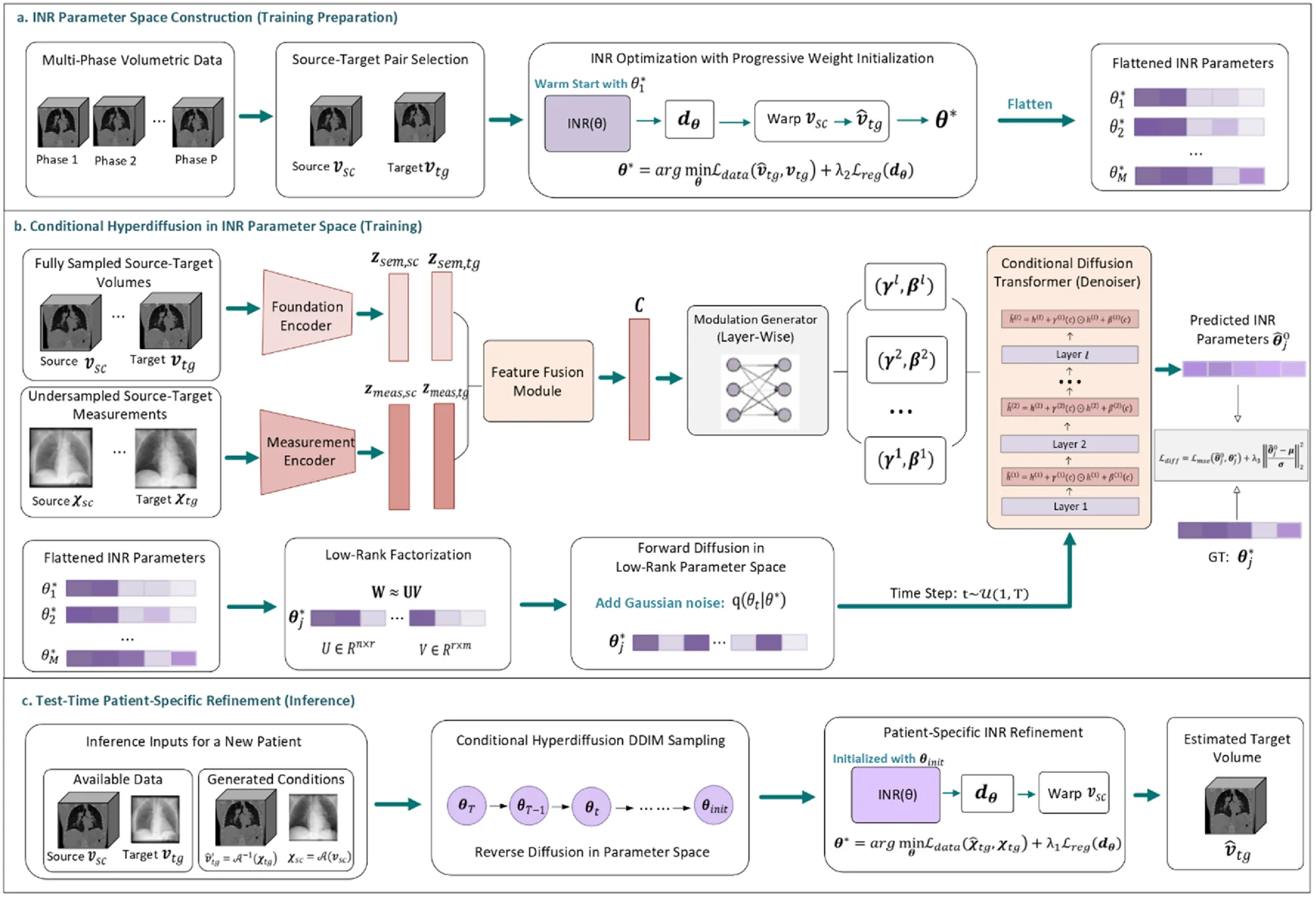
Overview of the proposed DiffINR framework. DiffINR consists of three stages: (a) offline INR parameter space (training set) construction from fully-sampled training volumetric image pairs, (b) conditional hyperdiffusion learning in INR parameter space using foundation model-derived semantic priors and measurement-aware features, and (c) test-time patient-specific refinement initialized from diffusion-predicted INR parameters for deformation-driven image estimation from limited measurements.

### 2.3. INR parameter space construction

In this stage, INRs were optimized for the training set $\left\{ {{P_i}} \right\}_{i = 1}^N$, where ${P_i}$ denotes a patient case with multiple volumetric images. For a given patient, multiple source-target pairs were constructed by alternately selecting one image as the source image ${v_{sc}}$ and using the other images as target images ${v_{tg}}$. For each source-target pair, an INR parameterized by ${\boldsymbol{\theta }}$ was optimized to represent the corresponding patient-specific DVF by minimizing a fully supervised, volumetric image-domain objective:

\begin{equation*}{\theta ^*} = {\mathrm{arg}}\mathop {\min }\limits_\theta {\mathcal{L}_{{\mathrm{data}}}}\left( {{{\hat v}_{{\mathrm{tg}}}},{ }{v_{{\mathrm{tg}}}}} \right) + {\lambda _2}{\mathcal{L}_{{\mathrm{reg}}}}\left( {{d_\theta }} \right),\end{equation*}

where ${\mathcal{L}_{data}}$ denotes the data fidelity loss between the deformed image ${\hat v_{{\mathrm{tg}}}}$ and the fully-sampled target image ${v_{tg}}$, and ${\lambda _2}$ balances the data fidelity objective and the regularization term. In contrast to the measurement-domain objective in equation (1), access to fully sampled training data enables offline optimization of high-quality INR parameters, which serve as reliable samples for subsequent diffusion learning.

By traversing all patients and patient-specific image pairs, a collection of optimized INR parameters $\left\{ {\boldsymbol{\theta }_j^{\mathrm{*}}} \right\}_{j = 1}^M$ was obtained. These optimized weights served as the ground truth for subsequent diffusion training. To encourage a smooth distribution in the INR parameter space, we adopted a progressive weight initialization strategy (ERKOç *et al* 2023), as shown in figure 1(a). Specifically, one set of INR parameters was first optimized for an initial training sample. The resulting optimized weights were then used to initialize the INR optimization for all subsequent training samples. This strategy promotes consistency across the learned parameter set and reduces abrupt parameter variations.

### 2.4. Conditional hyperdiffusion in INR parameter space

The above stage constructs an INR parameter space representing population-level deformation patterns that can be rapidly adapted to patient-specific parameters based on the limited target measurements (${\chi _{tg}}$) of a target volume. Accordingly, we developed a conditional hyperdiffusion model to generate informative initializations of patient-specific INR parameters. Specifically, each optimized INR parameter set ${\boldsymbol{\theta }}_j^{\mathrm{*}}$ in the population-based INR parameter space (Section 2.3), including all network weights and biases, was used as a training sample for conditional diffusion modeling. To reduce the parameter dimensionality, the weight matrices of the larger INR layers were represented using a rank*-r* low-rank factorization, ${\mathbf{W}} \approx {\mathbf{U}}{\boldsymbol{V}}$. This representation replaced each large parameter matrix with two smaller factor matrices, ${\mathbf{U}}$ and ${\boldsymbol{V}}$. The factorized parameters, together with the remaining unfactorized parameters, were then flattened and concatenated into a one-dimensional vector for diffusion modeling. For simplicity, the resulting compressed parameter vector is still denoted as ${\boldsymbol{\theta }}_j^{\mathrm{*}}$ below. Following prior studies (ERKOç *et al* 2023), a Transformer-based architecture was adopted as the denoising network of the conditional hyperdiffusion model to process these long parameter vectors. The hyperdiffusion training paradigm consisted of forward noising and reverse denoising processes over a multi-step diffusion chain. In the forward process, Gaussian noise was progressively added to the parameter vector ${\boldsymbol{\theta }}_j^{\mathrm{*}}$ at each diffusion step *t* to obtain a noisy sample ${\boldsymbol{\theta }}_j^t$. The noisy sample ${\boldsymbol{\theta }}_j^t$, together with the conditioning signals, was fed into the Transformer to predict ${\boldsymbol{\theta }}_j^0$, with ${\text{ }}{\boldsymbol{\theta }}_j^{\mathrm{*}}$ as the training target. The reverse process aimed to recover the clean parameters from the noisy input.

To facilitate patient-adaptive INR parameter generation, the Transformer was conditioned on two complementary features. The first condition consists of foundation model-derived semantic features extracted from the source and target volumes $\left( {{\boldsymbol{v}_{{\mathrm{sc}}}},{ }{\boldsymbol{v}_{{\mathrm{tg}}}}} \right)$, which were encoded by a pre-trained foundation encoder (Wang *et al* 2025) ${E_{{\mathrm{sem}}}}\left( \cdot \right)$, as shown in figure 1(b), to extract semantic features that provide anatomical deformation priors as ${\boldsymbol{z}_{{\mathrm{sem}}}} = {E_{{\mathrm{sem}}}}\left( {{\boldsymbol{v}_{{\mathrm{sc}}}},{ }{\boldsymbol{v}_{{\mathrm{tg}}}}} \right)$. Here, we directly used the fully sampled target image ${\boldsymbol{v}_{tg}}$, which is available from the training dataset, to construct ${\boldsymbol{z}_{{\mathrm{sem}}}}$. During inference (figure 1(c)), ${\boldsymbol{v}_{tg}}$ is the estimation target and is therefore unavailable. Instead, it is approximated by $\boldsymbol{\hat v}_{{\mathrm{tg}}}^{^{^{\prime}}}$, which is reconstructed from the limited measurements ${\boldsymbol{\chi} _{tg}}$ (section 2.5). The second condition consists of measurement-aware features encoded from paired limited source and target measurements $\left( {{\boldsymbol{\chi} _{{\mathrm{sc}}}},{ }{\boldsymbol{\chi} _{{\mathrm{tg}}}}} \right)$, where ${\boldsymbol{\chi} _{tg}}$ denotes the acquired target measurements and ${\boldsymbol{\chi} _{{\mathrm{sc}}}} = \mathcal{A}\left( {{\boldsymbol{v}_{{\mathrm{sc}}}}} \right)$ is generated from the fully sampled source image using the same acquisition operator, if not already available. A measurement encoder (figure 1(b)) used ${E_{{\mathrm{meas}}}}\left( \cdot \right)$ to capture raw measurement-based evidence by ${\boldsymbol{z}_{{\mathrm{meas}}}} = {E_{{\mathrm{meas}}}}\left( {{\boldsymbol{\chi} _{{\mathrm{sc}}}},{ }{\boldsymbol{\chi} _{{\mathrm{tg}}}}} \right)$. The two feature streams provided complementary guidance from complete anatomical structures and limited observations and were fused to form a latent conditional embedding $\boldsymbol{C}:$

\begin{equation*}\boldsymbol{C} = \phi \left( {\left[ {{\boldsymbol{z}_{{\mathrm{sem}}}},{ }{\boldsymbol{z}_{{\mathrm{meas}}}}} \right]} \right),\end{equation*}

where $\left[ {{\boldsymbol{z}_{{\mathrm{sem}}}},{ }{\boldsymbol{z}_{{\mathrm{meas}}}}} \right]$ denotes the concatenation of ${\boldsymbol{z}_{{\mathrm{sem}}}}$ and ${\boldsymbol{z}_{{\mathrm{meas}}}}$, and $\phi \left( \cdot \right)$ is a learnable feature fusion module.

Based on the fused embedding ${\boldsymbol{C}}$, a layer-wise residual modulation generator G (figure 1(b)) was used to generate adapted conditioning parameters for all Transformer blocks:

\begin{equation*}\left\{ {{\boldsymbol{\gamma }^{\left( l \right)}},{\boldsymbol{\beta }^{\left( l \right)}}} \right\}_{l = 1}^L = G\left( {\boldsymbol{C}} \right),\end{equation*}

where ${\boldsymbol{\gamma }^{\left( l \right)}}$ and ${\boldsymbol{\beta }^{\left( l \right)}}$ denote the layer-specific scaling and shifting parameters for the *l* th Transformer block. These parameters were used to modulate the hidden tokens ${\boldsymbol{h}^{\left( l \right)}}$:

\begin{equation*} {\boldsymbol{\widetilde h}^{\left( l \right)}} = {\boldsymbol{h}^{\left( l \right)}} + {\boldsymbol{\gamma} ^{\left( l \right)}} \odot {\boldsymbol{h}^{\left( l \right)}} + {\boldsymbol{\beta} ^{\left( l \right)}}\end{equation*}

where ⊙ denotes element-wise multiplication. By injecting these layer-specific modulation parameters throughout the denoising network, the Transformer was guided to generate anatomically plausible and measurement-consistent INR parameters.

The conditional hyperdiffusion model was optimized using a mean squared error loss between the predicted parameters $\widehat {\boldsymbol{\theta }}_j^0$ and the target parameters ${\text{ }}{\boldsymbol{\theta }}_j^{\mathrm{*}}$, together with a Gaussian prior regularization term,

\begin{equation*}{\mathcal{L}_{{\mathrm{diff}}}} = {\mathcal{L}_{{\mathrm{mse}}}}\left( {\hat {\boldsymbol{\theta}} _j^0,{ }{\boldsymbol{\theta}} _j^{\mathrm{*}}} \right) + {\lambda _3}\bigg\|\frac{{\hat \theta _j^0 - \mu }}{\sigma }\bigg\|_2^2,\end{equation*}

where ${\boldsymbol{\mu }}$ and ${\boldsymbol{\sigma }}$ denote the mean and standard deviation estimated from the optimized INR parameter space of the training set, and ${\lambda _3}$ balances the parameter-matching loss and the Gaussian prior regularization. This regularization term constrains the predicted INR weights to remain within the empirical distribution of optimized training INR parameters, thereby improving generation stability.

### 2.5. Test-time patient-specific refinement

During inference, a fully sampled source image ${\boldsymbol{v}_{sc}}$ and limited target measurements ${\boldsymbol{\chi} _{tg}}$ were available for a new patient. An approximate target volume $\boldsymbol{\widehat v}_{tg}^{\prime}$ was reconstructed from ${\boldsymbol{\chi} _{tg}}$ using a fast, conventional reconstruction method (for instance, Feldkamp-Davis-Kress (FDK) algorithm (Feldkamp *et al* 1984) for CBCT) for conditioning, denoted as $\boldsymbol{\hat v}_{{\mathrm{tg}}}^{^{^{\prime}}} = {\mathcal{A}^{ - 1}}\left( {{\boldsymbol{\chi} _{{\mathrm{tg}}}}} \right)$. These paired inputs $\left( {{\boldsymbol{v}_{{\mathrm{sc}}}},{ }\boldsymbol{\hat v}_{{\mathrm{tg}}}^{^{^{\prime}}}} \right)$ and $\left( {{\chi _{{\mathrm{sc}}}},{ }{\chi _{{\mathrm{tg}}}}} \right)$ were used as conditioning signals for the trained conditional hyperdiffusion model, which generated informative INR initialization parameters ${\theta _{{\mathrm{init}}}}$ from random noise through DDIM-based reverse sampling (Song *et al* 2020) in the INR parameter space.

Initialized with ${\boldsymbol{\theta} _{init}}$, the patient-specific INR predicted a dense DVF ${\boldsymbol{d}_{\boldsymbol{\theta }}}\left( {\boldsymbol{r}} \right)$, which was used to deform the source image ${\boldsymbol{v}_{sc}}$ through the spatial transformation operator $\mathcal{T}\left( \cdot \right)$, yielding the reconstructed target image ${\boldsymbol{\widehat v}_{tg}}$. The INR parameters were subsequently and quickly refined by minimizing the measurement-domain data fidelity objective defined in equation (1) using only the target measurements ${\boldsymbol{\chi} _{tg}}$.

### 2.6. Datasets

We evaluated the proposed DiffINR framework in two representative imaging applications with substantially different acquisition settings: limited-angle lung CBCT estimation and undersampled cardiac cine MRI estimation. The lung CBCT study represents an image-guided radiotherapy scenario in which a prior CT volume is available, and the current respiratory anatomy is estimated from limited-angle x-ray projections. The cardiac cine MRI study represents dynamic volumetric imaging in which a reference MRI volume is available, and the target cardiac motion state is estimated from highly undersampled k-space measurements. These applications involve different imaging modalities, measurement domains, acquisition processes, and motion patterns. The CBCT studies involved public and in-house datasets of lung patients (Castillo *et al* 2009, Vandemeulebroucke *et al* 2011, Hugo *et al* 2017, Shieh *et al* 2019). The MRI studies were performed on the publicly available OCMR cardiac cine MRI dataset (Chen *et al* 2020). In both studies, multi-motion-phase data were used to construct source-target volume pairs spanning different motion states; however, only two volumes are needed at a time for INR modeling, and each source-target pair is treated independently.

#### 2.6.1. Datasets for the limited-angle CBCT study

To train DiffINR (sections 2.3 and 2.4), we used a lung imaging database consisting of 26 patient cases (20 cases from The Cancer Imaging Archive (TCIA) (Hugo *et al* 2017) and 6 cases from the Creatis Laboratory (Vandemeulebroucke *et al* 2011)). Each case contains a 10-phase 4D-CT set, where all phase volumes were resampled to 256 × 256 × 256 with a voxel spacing of 1.5 × 1.5 × 1.5 mm^3^. Each phase was then alternately selected as the 3D source volume, while the remaining phases were used as target volumes, resulting in 90 fully sampled source-target pairs per case (2340 pairs in total). These pairs were used to optimize patient-specific INR models, yielding INR parameters that collectively formed the population-based parameter space (section 2.3) and served as ground-truth targets for hyperdiffusion training (section 2.4). The inputs for hyperdiffusion training include fully sampled source-target pairs and corresponding limited-angle measurements (figure 1(b)). To generate the inputs, limited-angle x-ray projections were retrospectively simulated from each fully sampled pair using a cone-beam forward projector. The scan angle extent was randomly selected (⩽90°), always starting from the anterior-posterior direction (0°). An orthogonal-view setting was used for training, by which projections were evenly distributed across two orthogonal angular spans, where the two views were separated by 90° and the reported scan extent denotes their combined angular coverage. The detector matrix was set to 256 × 256 with a pixel size of 1.5 × 1.5 mm^2^, while the source-to-isocenter and source-to-detector distances were 1000 and 1500 mm, respectively.

To evaluate the test-time refinement performance of DiffINR (section 2.5), we used two independent 4D-CT datasets and a clinical CBCT projection dataset. The first 4D-CT dataset was the DIR-Lab dataset (Castillo *et al* 2009), which comprises 10 patient cases, each with a 10-phase 4D-CT scan. The second was an in-house 4D-CT dataset (Werner *et al* 2009, Zhang *et al* 2016) consisting of 12 adult patients with lung tumors, each containing 10 or 14 respiratory-phase volumes. The 4D-CT images of the second dataset were acquired using a 16-slice Philips Brilliance CT scanner. The end-expiration (EE) phase was selected as the source volume, and the end-inspiration (EI) phase as the target volume, and the images were resampled to an isotropic voxel spacing of 1.5 × 1.5 × 1.5 mm^3^. Approximately 80 anatomical landmarks per patient were manually identified by a clinician at prominent pulmonary vascular and bronchial bifurcations on the EE and EI phases. The landmarks were distributed throughout the lungs, including the central lung regions, regions near the pleural boundaries, and regions near the tumor. The corresponding landmark pairs between the EE and EI phases were used to quantitatively evaluate the estimated DVFs. To mimic the clinical scenario where only a fully sampled source volume and limited-angle target projections are available, limited-angle source x-ray projections were generated from the fully sampled source volume, and limited-angle target projections were reconstructed into an approximated target volume to provide the inputs required for test-time refinement. The third dataset was from the SPARE challenge dataset (Shieh *et al* 2019), which uses clinically acquired cone-beam projections from five patient cases. Each case contains more than 1, 000 projection views in total, which were sorted into 10 respiratory phases. The 3D volume for each phase was reconstructed from the available projections (approximately 100 views per phase) using the FDK reconstruction algorithm (Feldkamp *et al* 1984). The reconstructed 50% respiratory phase was selected as the source volume, while the first 30 projections (covering approximately 0°-60°) of each remaining phase were used as the limited-angle target measurements.

We evaluated the robustness of DiffINR under different acquisition conditions at test time using the DIR-Lab dataset. Multiple limited-angle scan configurations were considered, including five scan angle extents (15°, 30°, 45°, 60°, and 90°) under two acquisition settings: single-view and orthogonal-view. In the single-view setting, projections were uniformly distributed within a single angular span, deviating from the orthogonal-view training setup. We further evaluated six angular offsets (0°, 30°, 60°, 90°, 120°, and 150°) for the starting direction of the limited scan angles for single-30°, ortho-30° (15° per view), single-90°, and ortho-90° (45° per view) scenarios. These settings (Qian *et al* 2025) were selected to characterize the performance of DiffINR under progressively reduced angular coverage and variations in acquisition geometry. A contiguous single-view span is more directly compatible with a shortened conventional gantry rotation, whereas the orthogonal-view setting evaluates complementary projection information from two angular ranges separated by 90° (Ren *et al* 2016). The angular-offset study further assesses robustness to variations in the starting and ending angles of limited-angle acquisitions (Zhang *et al* 2017).

The performance of DiffINR was quantitatively evaluated in terms of image similarity and registration accuracy (Qian *et al* 2025). Structural similarity index (SSIM) (Wang *et al* 2004) and relative error (RE) (Li and Xing 2007) were used to assess the similarity between the estimated limited-angle CBCTs and the reference volumes, while target registration error (TRE) (Castillo *et al* 2009) was used to evaluate DVF accuracy by calculating the Euclidean distance between DVF-propagated lung landmarks and the corresponding expert-annotated landmarks.

#### 2.6.2. Dataset for the real-time volumetric MRI study

The MRI experiments were performed using the publicly available OCMR dataset (Chen *et al* 2020) to evaluate DiffINR. The MR scans were acquired on three Siemens Magnetom scanners: Prisma (3 T), Avanto (1.5 T), and Sola (1.5 T) (Siemens Healthineers, Munich, Germany), using a gradient-echo steady-state free precession sequence. The dataset contains diverse acquisition settings, including variations in magnetic field strength, spatial resolution, and field of view. The images exhibit mixed T2/T1 contrast, with repetition time ranging from 28.5 to 39.3 ms, echo time from 1.41 to 1.53 ms, and flip angles between 33° and 70°.

To establish high-quality reference volumes for quantitative evaluation, 11 fully sampled subjects were included in this study. For each subject, volumetric cine cardiac images were acquired in planar mode, with short-axis slices spanning from the base to the apex of the heart. Each cardiac cycle was divided and sorted into multiple temporal bins, where the 0% bin corresponds to the end-diastolic state. Complex-valued MR images were reconstructed from the fully sampled k-space data using the nonuniform fast Fourier transform (NUFFT) algorithm (Fessler and Sutton 2003). The reconstructed volumes were resampled to 256 ×256 ×32 using trilinear interpolation, and voxel values were further normalized such that the maximum absolute voxel value across the real and imaginary parts of a complex-valued volume is one. A five-fold cross-validation strategy was adopted, with nine subjects used for training and two for testing in each fold, yielding approximately 209–215 cardiac bins for training and 43–49 bins for testing (Qian *et al* 2026). During training, each cardiac bin of a subject was alternately selected as the source volume, with all other bins of the same subject treated as target volumes. For testing, the first cardiac bin (0%) of each subject was used as the source volume, and all other bins of the same subject served as target volumes. Undersampled k-space data with 13 golden-angle radial spokes per slice were retrospectively generated from the fully sampled images via NUFFT to provide limited-measurement inputs for hyperdiffusion training and testing. To further evaluate the robustness under different sampling conditions, additional experiments were conducted using 9 and 5 spokes per slice during test time.

The performance of volumetric MRI estimation was quantitatively evaluated using two image-quality metrics and three contour-based measures (Qian *et al* 2026). Specifically, SSIM and RE were used to assess the similarity between the estimated and reference volumes. In addition, Dice similarity coefficient (Dice) (MüLLER *et al* 2022), 95th percentile Hausdorff distance (HD95) (Taha and Hanbury 2015), and center-of-mass error (COME) (Shao *et al* 2022) were calculated between manually delineated left ventricle contours and those propagated from source volumes by DVFs, to evaluate the DVF accuracy.

### 2.7. Implementation details

We used the pretrained encoder (${E_{sem}}\left( \cdot \right)$) of a foundation model SAM-Med3D (Wang *et al* 2025) to extract semantic features from fully-sampled imaging volume pairs to serve as the deformation priors (Sec. 2.4), which remained frozen during training. A 384-dimensional embedding was generated for each input volume. In parallel, the other measurement encoder (${E_{meas}}\left( \cdot \right)$) consisted of three stacked 1D/2D convolutional blocks, followed by global average pooling and two fully connected layers, producing a 512-dimensional feature vector from limited measurements. The image-domain and measurement-domain embeddings were concatenated and passed through the two-layer MLP feature fusion module (ϕ, equation (3)) with a hidden dimension of 512 to generate the fused condition embedding. This embedding was then fed into the modulation generator (G, equation (4)) to predict the layer-wise modulation parameters for the denoising network (figure 1(b)). The denoising backbone was implemented as a six-layer Transformer with a token dimension of 512 and 8 attention heads.

The DVF was represented by a Siren-based INR (Sitzmann *et al* 2020) with layer dimensions of (3, 256, 256, 256, 3). To reduce the computational burden of performing diffusion over high-dimensional INR parameters, low-rank factorization was applied to the hidden-layer weight matrices. Specifically, the two largest 256 ×256 weight matrices were factorized into pairs of low-rank matrices of 256 ×32 and 32 ×256. Diffusion was then performed in the resulting compressed parameter space, as illustrated in figure 1(b). In addition, since INR parameters have relatively small numerical ranges compared with standard Gaussian diffusion noise, dataset-level mean and standard deviation statistics were computed from all optimized INR parameters in section 2.3. Z-score normalization was then applied to the optimized INR parameters before training the hyperdiffusion model in section 2.4. The parameters sampled from the hyperdiffusion model were restored to the original scale before test-time refinement in section 2.5.

For INR optimization, Adam was used with a learning rate of 5×10^−5^. For the progressive weight initialization strategy applied for INR parameter space construction (section 2.3), the first case was optimized for 1000 epochs, while subsequent cases were initialized from the optimized weights of the first case and optimized for 200 epochs to promote INR parameter consistency, reduce abrupt variations, and encourage a smooth diffusion process (section 2.4) over the set of optimized INR weights (ERKOç *et al* 2023). For hyperdiffusion training, the AdamW optimizer was used with a learning rate of 1×10^−5^ for 6000 epochs, and the batch size was set to 32. The forward diffusion process used 1000 timesteps with a cosine noise schedule, while 200 timesteps were used for DDIM sampling during inference. During the test-time refinement, 50 INR optimization epochs were performed for each case. The regularization weight in equation (6) for hyperdiffusion training was set to 1 × 10^−3^, and the DVF regularization weights in equations (1) and (2) were both set to 0.05. The data-fidelity term used the L2 loss for MRI and the L1 loss for CBCT experiments.

### 2.8. Evaluation protocols

#### 2.8.1. Ablation settings

To validate the key components of the proposed DiffINR framework, we designed three ablation studies on the DIR-Lab (for limited-angle CBCT) and OCMR (for real-time MRI) testing datasets. The first study investigated the effect of INR parameter space construction for subsequent hyperdiffusion training. Two strategies were compared: (1) parameter space construction from fully sampled images, where optimized INR parameters were obtained using fully sampled, high-quality target volumes for supervision (our DiffINR strategy); and (2) parameter construction from limited measurements, where INR parameters were optimized by minimizing the data fidelity loss in the projection or k-space domain (equation (1)). The second study evaluated the contribution of conditional inputs used in hyperdiffusion model training. Four settings were considered: (1) volume-only conditioning, using fully sampled image-volume priors without measurement inputs; (2) measurement-only conditioning, using limited measurements without image-volume priors; (3) dual-stream conditioning with an approximated target volume, where both image-domain priors and measurement-domain observations were jointly used but the target volume used for image-domain conditioning was reconstructed from limited measurements; and (4) dual-stream conditioning with the fully sampled target volume (our DiffINR strategy). Note that the differentiations between fully sampled or approximated target volumes only apply to hyperdiffusion training; at test time, only the approximated target volumes are available. The third study assessed the role of hyperdiffusion-based initialization and test-time refinement by comparing three variants: (1) INR-only, where the INR was randomly initialized and optimized for 2000 epochs; (2) hyperdiffusion-only, where the INR parameters generated by conditional DDIM sampling from the hyperdiffusion model were directly used as the final output; and (3) hyperdiffusion initialization with INR refinement, where the initialized INR parameters were further optimized for 50 epochs in a patient-specific manner (our DiffINR strategy).

#### 2.8.2. Hyperparameter analysis settings

We analyzed the effects of epoch number, learning rate, and DVF regularization weight used in test-time refinement on the performance of DiffINR. For the hyperparameter analysis, the limited-angle CBCT experiments were performed on the DIR-Lab dataset under the ortho-90° limited-angle setting, and the real-time volumetric MRI experiments were performed on the OCMR dataset using 13-spoke k-space sampling. For refinement epoch numbers, we compared five epoch settings (30, 50, 100, 150, 200), to evaluate the trade-off between runtime and performance improvement. For the learning rate of the INR optimizer, four candidate values of (1 × 10^−4^, 5 × 10^−4^, 1 × 10^−5^, 5 × 10^−5^) were tested to identify a stable update step size. We further examined the regularization weight ${{{\lambda }}_1}$ in equation (1) for test-time INR optimization, which controls the smoothness of the estimated DVFs. Five values (0, 0.02, 0.05, 0.08, and 0.1) were evaluated to investigate the effect of smoothness regularization on DVF flexibility and regularity.

#### 2.8.3. Comparison settings

Since DiffINR formulates volumetric image estimation as a registration-driven problem, the comparison focused on deformation-based estimation methods. We compared the proposed DiffINR with representative methods spanning different methodological categories, including conventional iterative optimization, population-based one-time inference, test-time optimization, and hybrid methods.

1) Iterative (Zhang *et al* 2013b): a conventional registration-driven image estimation method that directly optimizes the DVF for each case using gradient-based updates.
2) RegNet (Zhang 2021): a registration network trained on a population-based cohort and directly applied to the testing cases without case-specific adaptation.
3) RegNet-FT (Zhang 2021): a test-time adaptation variant of RegNet. The pretrained RegNet was further fine-tuned for each testing case for 2000 epochs to enhance patient-specific performance.
4) RegNet-DVF (Miura *et al* 2024): a population-based method with a two-step training strategy: a 3D registration network was first trained on 3D image pairs to predict DVFs, which were then used as regularization to guide limited-measurement-based registration optimization.
5) RegNet-Gen (Dong *et al* 2023): a reconstruction-assisted registration method in which a generator was introduced to estimate a volumetric image from limited measurements, followed by volume-to-volume deformable registration.
6) INR (Wolterink *et al* 2022): a patient-specific INR baseline optimized from scratch for each testing case for 2000 epochs, without learned initialization or population-level priors.
7) Hb-2D3DReg (Qian *et al* 2025): a hybrid framework for limited-angle CBCT estimation that combines population-level deformation priors with patient-specific INR refinement. The population-based model provides a coarse deformation initialization, which is then refined by an INR during test-time optimization.
8) cMeta-INR (Qian *et al* 2026): a meta-learning-based INR framework for real-time volumetric MRI estimation that learns transferable initialization parameters for rapid patient-specific adaptation.

All comparison methods were implemented under the same hardware/software settings for fair evaluation. For each comparison method, the hyperparameter settings and optimization protocol followed the corresponding original formulation or recommended configuration. Test-time efficiency was evaluated on an Nvidia H100 GPU using the DIR-Lab dataset under the ortho-90° setting (45° per view) for CBCT and the OCMR dataset with 13 radial spokes per slice for MR. Runtime was recorded per testing case and included the complete test-time pipeline, including initialization inference, where applicable, and patient-specific refinement. Statistical comparisons were performed using Wilcoxon signed-rank tests with Bonferroni correction between DiffINR and each comparison method or ablation variant for each metric. Corrected *p-*values below 0.05 were considered statistically significant.

## 3. Results

We first conducted ablation and hyperparameter analyses to assess the effects of key components and parameter settings of DiffINR (sections 3.1 and 3.2). DiffINR was then compared with representative methods on both CBCT and MRI estimation tasks (Section 3.3). Finally, its robustness and computational efficiency were analyzed in section 3.4.

### 3.1. Ablation study

As shown in tables 1 and 2, compared with the variant that uses an INR parameter space constructed from limited measurements, the full DiffINR setting achieved consistently better image quality and DVF accuracy metrics. This indicates that a high-quality INR parameter space derived from fully sampled image pairs enabled more reliable diffusion learning of the INR parameter distribution, thereby improving subsequent test-time optimization and volumetric image estimation. For the conditioning strategy, both the volume-only and measurement-only variants yielded inferior performance compared with DiffINR, indicating that image-domain anatomical priors and measurement-domain observations provide complementary information for conditional diffusion learning and patient-specific INR optimization. The dual-stream variant conditioned on target volumes approximated from limited measurements generally outperformed the two single-stream variants, further supporting the benefit of combining both conditioning sources. However, it remained inferior to DiffINR, whose hyperdiffusion model was trained using fully sampled target volumes. Although only target volumes approximated from limited measurements were available at test time for all methods, the population-based hyperdiffusion training in DiffINR leveraged foundation-model features extracted from high-quality, fully sampled source-target volume pairs to learn more robust anatomical priors. These priors provided a stronger initialization for subsequent patient-specific test-time refinement. Results of the INR-only variant indicated that random initialization was insufficient for rapid patient-specific adaptation, requiring substantially more test-time optimization (2000 epochs vs. 50 epochs for DiffINR) while still yielding inferior performance. Meanwhile, the results of the hyperdiffusion-only variant suggested that the diffusion model-generated parameters provided an informative initialization while requiring further test-time refinement.

**Table 1.** Comparison of mean (±s.d.) SSIM, RE, and TRE metrics in the ablation study on the **DIR-Lab** testing dataset for limited-angle CBCT estimation. Testing was performed under the ortho-90° (45° each) limited-angle scenario, with the orthogonal angles starting from the anterior-posterior direction (0°) and the left-lateral direction (90°) of the scanned subject. DiffINR denotes the full model using fully-sampled volumetric image pairs for INR parameter space construction, dual-stream (image volume-based and measurement-based) conditioning, and hyperdiffusion-based initialization followed by test-time INR refinement. Wilcoxon signed-rank tests with Bonferroni corrections were performed between DiffINR and the ablation variants for each metric, with all corrected *p*-values below 0.05. The arrows are pointing in the direction of improved accuracy. Best values are marked in **bold**.

Ablation study /DIR-Lab	Variant	Mean (± s.d.)		
		SSIM↑	RE/%↓	TRE/mm↓
INR parameter space construction	From limited measurements	0.958 ± 0.022	7.02 ± 2.26	3.97 ± 2.16

Conditioning	Volume-only	0.953 ± 0.028	7.34 ± 2.32	4.52 ± 2.11
	Measurement-only	0.956 ± 0.036	7.16 ± 2.15	4.21 ± 2.40
	Dual-stream (approximated target)	0.959 ± 0.028	7.03 ± 2.04	3.89 ± 1.97

Refinement	Hyperdiffusion-only	0.938 ± 0.034	10.08 ± 1.03	6.14 ± 2.28
	INR-only	0.949 ± 0.035	9.71 ± 2.33	5.01 ± 2.77

Full model	DiffINR	**0.962 ± 0.021**	**6.84 ± 1.70**	**3.61 ± 1.27**

**Table 2.** Comparison of mean (±s.d.) SSIM, RE, Dice, HD95, and COME metrics in the ablation study on the **OCMR** testing dataset for real-time volumetric MRI estimation. The testing was based on sparse sampling of 13 k-space radial spokes per slice. Wilcoxon signed-rank tests with Bonferroni corrections were performed between DiffINR and the ablation variants for each metric, with all corrected *p*-values below 0.05.

Ablation study / OCMR	Variant	Mean (± s.d.)				
		SSIM↑	RE/%↓	Dice↑	HD95↓	Come/mm↓
INR parameter space construction	From limited measurements	0.982 ±0.007	8.32 ±3.28	0.87 ±0.05	4.42 ±2.06	1.54 ±1.02

Conditioning	Volume-only	0.979 ±0.007	8.75 ±3.12	0.86 ±0.05	4.96 ±1.88	1.72 ±1.17
	Measurement-only	0.980 ±0.006	8.58 ±2.79	0.86 ±0.04	4.83 ±1.38	1.68 ±1.15
	Dual-stream (approximated target)	0.982 ±0.007	8.34 ±2.20	0.88 ±0.06	4.31 ±1.61	1.45 ±1.03

Refinement	Hyperdiffusion-only	0.962 ±0.009	10.06 ±3.83	0.83 ±0.07	5.35 ±2.00	2.11 ±1.26
	INR-only	0.976 ±0.008	8.78 ±3.28	0.86 ±0.05	4.72 ±1.53	1.74 ±1.18

Full model	DiffINR	**0.985 ±0.007**	**8.03 ±2.16**	**0.89 ±0.04**	**4.06 ±1.33**	**1.30 ±1.09**

### 3.2. Hyperparameter analysis

Figures 2 and 3 show the hyperparameter analyses of DiffINR on the limited-angle CBCT and real-time volumetric MRI estimation tasks, respectively. Increasing the number of INR refinement epochs generally improved performance, with progressively smaller gains after 50 epochs (figures 2(a) and 3(a)). DiffINR also showed relatively robust performance across the tested INR learning rates (figures 2(b) and 3(b)), with a learning rate of 5×10^−5^ providing the overall best performance for both testing sets. Similarly, when varying the smoothness regularization weight ${\lambda _1}$, a value of 0.05 provided the best overall performance (figures 2(c) and 3(c)). Based on the results of both studies (CBCT/MRI), 50 refinement epochs, a learning rate of 5 × 10^−5^, and ${\lambda _1}$ = 0.05 were adopted as the default settings for subsequent experiments.

**Figure 2.**
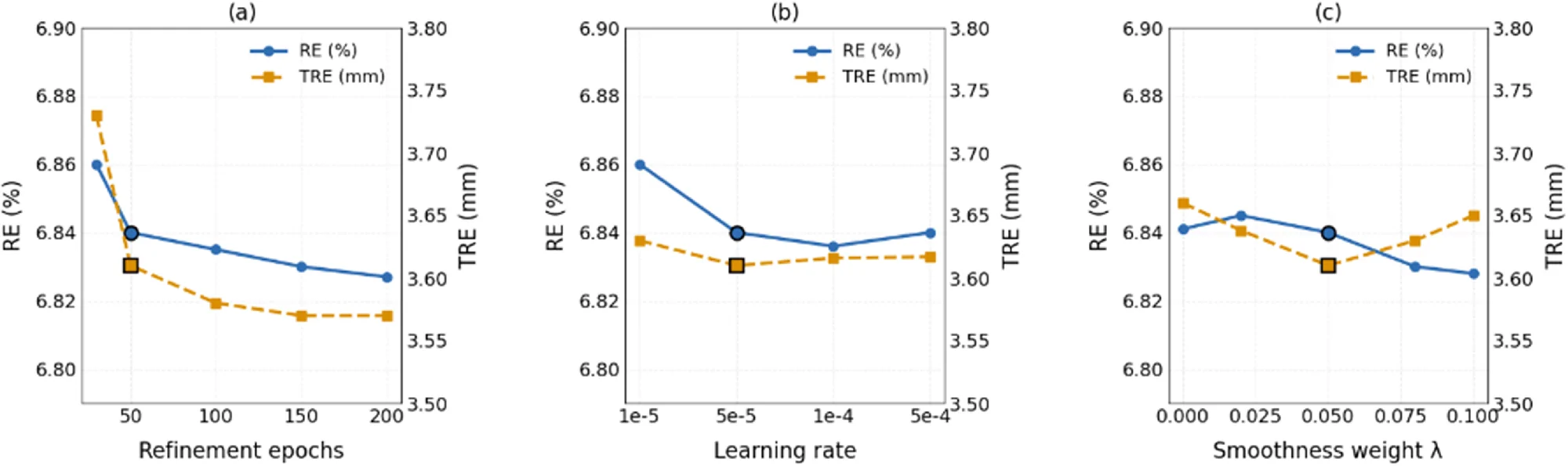
Hyperparameter analysis of DiffINR on the DIR-Lab dataset for: (a) Number of refinement epochs, (b) Optimizer learning rate, and (c) Smoothness regularization weight ${\lambda _1}$. RE and TRE were reported to evaluate the image accuracy and DVF accuracy, respectively. The analysis was performed under the ortho-90° limited-angle setting (45° per view), with the two orthogonal views starting at 0° (anterior-posterior) and 90° (left-lateral) directions.

**Figure 3.**
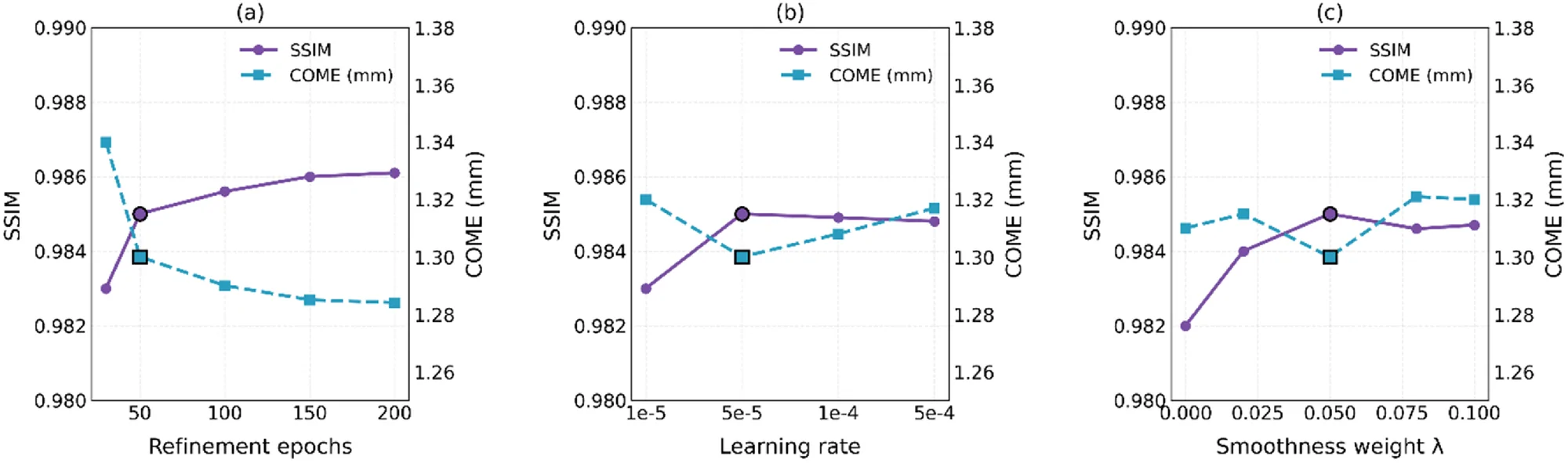
Hyperparameter analysis of DiffINR on the **OCMR** dataset for: (a) Number of refinement epochs, (b) Optimizer learning rate, and (c) Smoothness regularization weight ${\lambda _1}$. SSIM and COME were used to evaluate the image accuracy and DVF accuracy, respectively. All analyses were based on 13 radial spokes per slice.

### 3.3. Comparison with other methods

#### 3.3.1. CBCT results

##### 3.3.1.1. Quantitative comparison of different methods on three datasets

Tables 3- 5 quantitatively compare different methods via studies on three datasets. Overall, DiffINR achieved the best or comparable performance across all datasets, supporting its effectiveness and robustness for limited-angle CBCT estimation on both simulated projection data (DIR-Lab and in-house datasets) and clinically acquired CBCT projection data (Spare projection dataset). These results suggest that the diffusion-generated initialization effectively guided patient-specific refinement and improved limited-angle CBCT estimation accuracy. The Bonferroni-corrected Wilcoxon signed-rank tests showed that most pairwise comparisons between DiffINR and the other methods were statistically significant across the three CBCT datasets, indicating generally consistent improvements in image estimation and registration accuracy.

**Table 3.** Comparison of mean (± s.d.) SSIM, RE, and TRE metrics across nine methods on the **DIR-Lab** testing dataset. Testing was performed under the ortho-90° limited-angle setting (45° per view), with the two orthogonal views starting at 0° (anterior–posterior) and 90° (left-lateral) directions. Wilcoxon signed-rank tests with Bonferroni corrections were performed between DiffINR and each competing method except zero-DVF.

Methods/DIR-Lab	Mean (± s.d.)			*p*-value		
	SSIM↑	RE/%↓	TRE/mm↓	SSIM	RE	TRE
Zero-DVF	0.925 ± 0.035	15.40 ± 2.41	8.52 ± 3.31	—	—	—
Iterative	0.946 ± 0.040	8.85 ± 2.34	5.02 ± 2.37	0.007	0.006	0.003
RegNet	0.942 ± 0.034	9.82 ± 2.12	6.38 ± 2.46	0.006	0.002	0.005
RegNet-FT	0.950 ± 0.024	8.73 ± 1.98	4.94 ± 2.35	0.009	0.009	0.011
RegNet-DVF	0.945 ± 0.035	9.02 ± 2.62	5.35 ± 2.72	0.007	0.003	0.003
RegNet-Gen	0.948 ± 0.031	8.97 ± 2.54	5.11 ± 2.13	0.005	0.006	0.006
INR	0.949 ± 0.035	9.71 ± 2.33	5.01 ± 2.77	0.007	0.009	0.003
Hb-2D3DReg	0.953 ± 0.035	7.99 ± 2.16	3.70 ± 1.94	0.045	0.052	0.074
DiffINR	**0.962 ± 0.021**	**6.84 ± 1.70**	**3.61 ± 1.27**	**—**	**—**	**—**

**Table 4.** Comparison of mean (± s.d.) SSIM, RE, and TRE metrics across nine methods on the **in-house** testing dataset. Testing was performed under the ortho-90° limited-angle setting (45° per view), with the two orthogonal views starting at 0° (anterior–posterior) and 90° (left-lateral) directions. Wilcoxon signed-rank tests with Bonferroni corrections were performed between DiffINR and each competing method except zero-DVF.

Methods/in-house	Mean (± s.d.)			*p*-value		
	SSIM↑	RE/%↓	TRE/mm↓	SSIM	RE	TRE
Zero-DVF	0.875 ± 0.041	18.24 ± 2.48	6.69 ± 1.76	—	—	—
Iterative	0.918 ± 0.085	13.14 ± 3.45	3.87 ± 1.56	0.051	0.038	0.024
RegNet	0.905 ± 0.032	14.97 ± 2.09	5.03 ± 2.53	0.008	0.007	0.006
RegNet-FT	0.910 ± 0.070	13.67 ± 4.02	4.83 ± 1.48	0.009	0.010	0.007
RegNet-DVF	0.912 ± 0.025	14.35 ± 2.18	5.00 ± 1.71	0.011	0.008	0.007
RegNet-Gen	0.916 ± 0.022	13.64 ± 2.35	4.88 ± 1.63	0.024	0.025	0.009
INR	0.903 ± 0.083	14.09 ± 4.48	4.05 ± 1.43	0.009	0.009	0.011
Hb-2D3DReg	0.920 ± 0.036	**12.98 ± 3.96**	**3.12 ± 0.94**	0.055	1.000	0.382
DiffINR	**0.928 ± 0.021**	12.99 ± 1.76	3.14 ± 0.76	—	—	—

**Table 5.** Comparison of mean (± s.d.) SSIM and RE metrics across nine methods on the **SPARE projection** dataset. Testing was performed using single-view cone-beam projections with an average angular coverage of approximately 60°. Wilcoxon signed-rank tests with Bonferroni corrections were performed between DiffINR and each competing method except zero-DVF.

Methods/spare projection	Mean (± s.d.)		*p*-value	
	SSIM↑	RE/%↓	SSIM	Re
Zero-DVF	0.816 ± 0.039	17.20 ± 6.05	—	—
Iterative	0.848 ± 0.062	15.24 ± 5.65	0.004	0.006
RegNet	0.828 ± 0.055	16.54 ± 4.24	<0.001	<0.001
RegNet-FT	0.833 ± 0.071	15.98 ± 5.66	<0.001	<0.001
RegNet-DVF	0.835 ± 0.055	15.65 ± 4.89	<0.001	0.002
RegNet-Gen	0.839 ± 0.073	15.37 ± 3.52	<0.001	0.004
INR	0.841 ± 0.067	15.16 ± 3.63	0.002	0.003
Hb-2D3DReg	0.866 ± 0.048	14.12 ± 4.58	0.008	0.009
DiffINR	**0.873 ± 0.049**	**13.75 ± 3.02**	**—**	**—**

##### 3.3.1.2. Visual comparison of different methods on three datasets

Figures 4–6 show the visual comparison of different methods on the DIR-Lab, in-house, and SPARE projection datasets. The visual results were consistent with the quantitative results. Without registration (zero-DVF), noticeable anatomical mismatch was observed between the source and target volumes, leading to substantial residual errors in the difference maps. Population-based methods, including RegNet and its variants, reduced the mismatch but still showed local residuals, particularly around high-contrast anatomical boundaries and regions with large deformation. INR-based patient-specific optimization further improved anatomical alignment; however, residual discrepancies remained in some local structures. Compared with these methods, DiffINR generated estimated CBCT volumes that were visually closer to the target-GT across all three views. The zoom-in regions further demonstrated that DiffINR better recovered local anatomical details.

**Figure 4.**
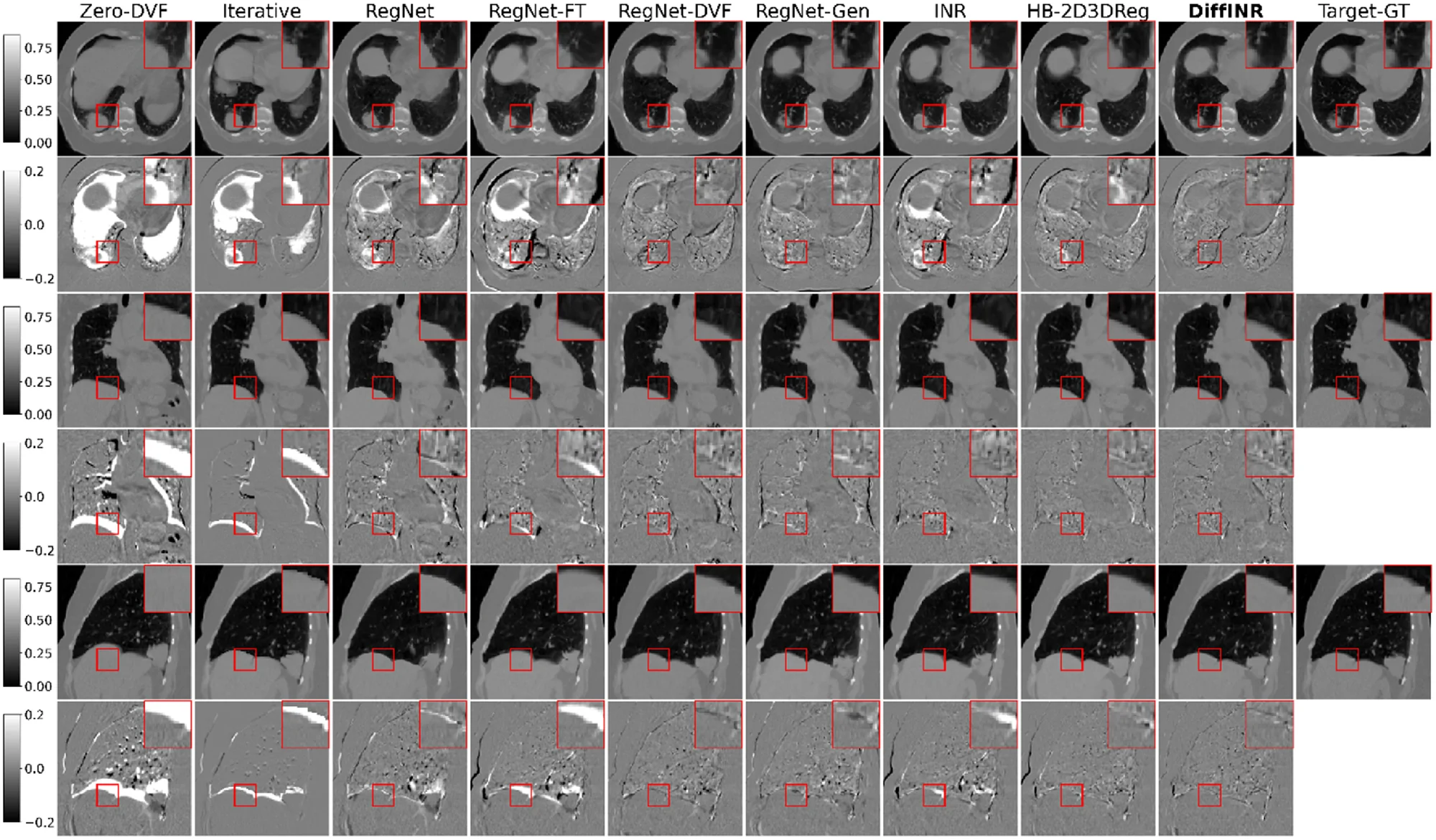
Three-view comparison of estimated limited-angle CBCT volumes (odd rows) and corresponding difference maps (even rows) across nine methods for a patient case from the **DIR-Lab** dataset. Target-GT represents the ground-truth target volume. The testing settings follow those described in table 3. A zoom-in inset (top-right) is provided to show local details.

**Figure 5.**
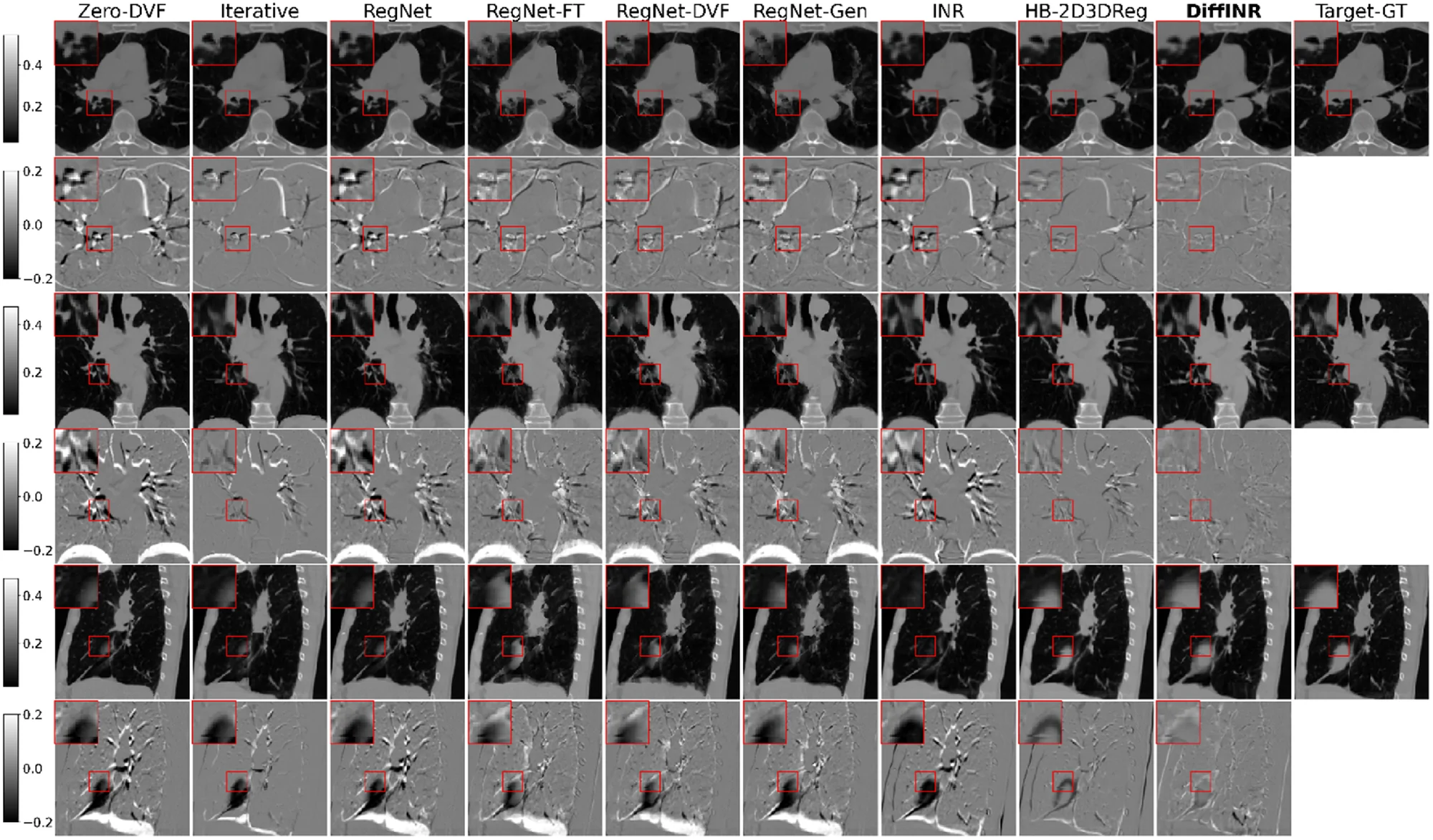
Three-view comparison of estimated limited-angle CBCT volumes (odd rows) and corresponding difference maps (even rows) across nine methods for a patient case from the **in-house** dataset. Target-GT represents the ground-truth target volume. The testing settings follow those described in table 4. A zoom-in inset (top-left) is provided to show local details.

**Figure 6.**
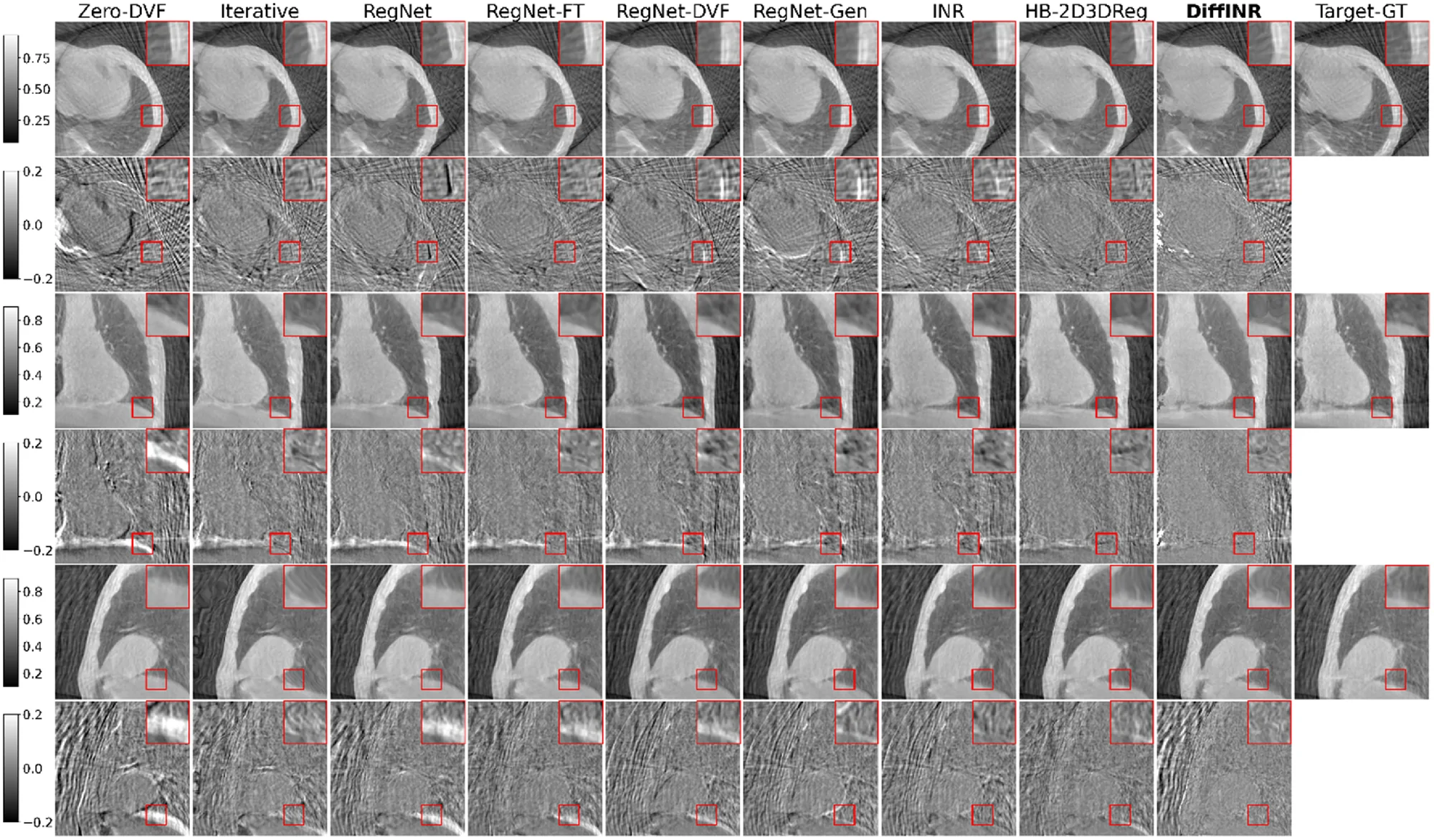
Three-view comparison of estimated limited-angle CBCT volumes (odd rows) and corresponding difference maps (even rows) across nine methods for a patient case from the **SPARE projection** dataset. Target-GT represents the ‘ground-truth’ target volume. The testing settings follow those described in table 5. A zoom-in inset (top-right) is provided to show local details.

#### 3.3.2. MRI results

##### 3.3.2.1. Quantitative comparison of different methods on the MRI dataset

Table 6 compares the quantitative results of different methods on the OCMR dataset. DiffINR achieved competitive overall performance in terms of SSIM, Dice, and COME, with values of 0.985 ± 0.007, 0.89 ± 0.04, and 1.30 ± 1.09 mm, respectively. Compared with the conventional Iterative method and INR optimized from scratch, DiffINR improved both image-estimation accuracy and DVF accuracy, demonstrating the benefit of diffusion-based initialization for test-time refinement. Compared with population-based methods, including RegNet and its variants, DiffINR achieved higher SSIM and Dice and lower COME, reflecting better generalization to unseen testing cases. Although cMeta-INR achieved the lowest RE and HD95, DiffINR obtained better SSIM, lower COME, and comparable Dice. In general, DiffINR effectively combined diffusion model-generated INR parameter priors with test-time adaptation for accurate volumetric MRI estimation from undersampled k-space data. The Bonferroni-corrected Wilcoxon analysis further revealed statistically significant differences between DiffINR and most comparison methods.

**Table 6.** Comparison of mean (± s.d.) SSIM, RE, Dice, HD95, and COME across nine methods on the **OCMR** dataset for real-time volumetric MRI estimation. The testing was based on sparse sampling of 13 k-space radial spokes per slice. Wilcoxon signed-rank tests with Bonferroni corrections were performed between DiffINR and each competing method except zero-DVF.

Methods	Mean (± s.d.)					*p*-value				
	SSIM↑	Re/%↓	Dice↑	HD95↓	Come/mm↓	SSIM	RE	Dice	HD95	COME
Zero-DVF	0.956 ± 0.007	13.45 ± 4.46	0.77 ± 0.12	6.24 ± 2.58	2.47 ± 1.66	—	—	—	—	—
Iterative	0.979 ± 0.009	8.86 ± 4.13	0.86 ± 0.02	4.54 ± 1.82	1.57 ± 1.28	0.003	<0.001	<0.001	0.002	<0.001
RegNet	0.976 ± 0.007	9.64 ± 4.21	0.87 ± 0.05	4.65 ± 1.48	1.71 ± 1.33	<0.001	<0.001	<0.001	<0.001	<0.001
RegNet-Ft	0.979 ± 0.009	9.00 ± 3.57	0.87 ± 0.07	4.53 ± 1.92	1.58 ± 1.25	<0.001	<0.001	<0.001	<0.001	<0.001
RegNet-DVF	0.972 ± 0.006	8.34 ± 4.37	0.85 ± 0.06	5.01 ± 2.05	1.83 ± 1.53	<0.001	0.003	<0.001	<0.001	<0.001
RegNet-Gen	0.978 ± 0.007	8.26 ± 4.12	0.87 ± 0.06	4.49 ± 1.38	1.50 ± 1.06	<0.001	0.008	0.002	0.003	<0.001
INR	0.976 ± 0.008	8.78 ± 3.28	0.86 ± 0.05	4.72 ± 1.53	1.74 ± 1.18	<0.001	<0.001	<0.001	0.002	<0.001
cMeta-INR	0.983 ± 0.007	**7.44 ± 2.76**	0.89 ± 0.07	**3.53 ± 1.26**	1.32 ± 1.02	0.042	0.031	1.000	0.263	0.050
DiffINR	**0.985 ± 0.007**	8.03 ± 2.16	**0.89 ± 0.04**	4.06 ± 1.33	**1.30 ± 1.09**	—	—	—	—	—

##### 3.3.2.2. Visual comparison of different methods on the MRI dataset

Figure 7 visually compares different methods on a real-time volumetric MRI estimation case. The visual results corroborated the quantitative results in table 6, with DiffINR showing an estimated MRI volume matching best with the ground-truth target volume.

**Figure 7.**
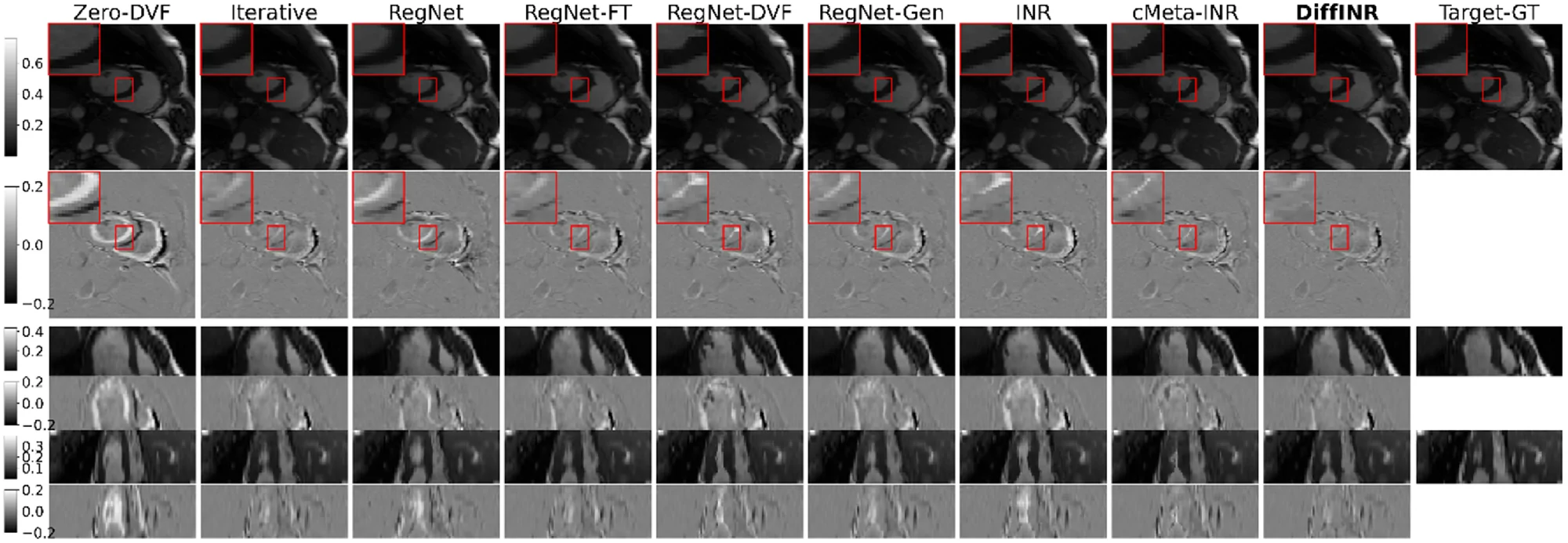
Three-view comparison of estimated real-time volumetric MRI images (odd rows) and corresponding difference maps (even rows) across nine methods for a subject from the **OCMR** dataset. Target-GT represents the ground-truth target volume. The testing settings follow those described in table 6. A zoom-in inset (top-right) is provided to show local details.

### 3.4. Robustness and efficiency analysis

#### 3.4.1. Robustness under different sampling conditions

Tables 7 and 8 present the robustness evaluation of DiffINR under different limited-angle CBCT acquisition settings on the DIR-Lab dataset. As shown in table 7, DiffINR maintained stable performance across five scan-angle magnitudes under both single-view and orthogonal-view acquisition scenarios. As the scan angle increased from 15° to 90°, the image-estimation and DVF accuracy generally improved, which is expected as larger scan-angle coverage provided more projection information for constraining the patient-specific refinement. Under the same scan-angle magnitude, the orthogonal-view setting achieved better performance than the single-view setting, indicating that complementary angular information from orthogonal views was beneficial for limited-angle CBCT estimation. Table 8 further evaluated the sensitivity of DiffINR to different starting angle offsets. Across the tested 30° and 90° settings, the performance variations were limited, demonstrating that DiffINR was robust to scan-direction changes not included in training. Clinically, the feasible angular coverage depends on acquisition speed, anatomical motion, mechanical constraints from the treatment geometry or patient-support devices, and the imaging system (Zhang *et al* 2013a). Larger angular coverage generally provides more projection information but may require longer acquisition times, increase motion blurring, and face mechanical clearance challenges. A shortened single-view acquisition may therefore be easier to integrate into conventional gantry-based workflows, while separated angular ranges may be advantageous when supported by the imaging system. The preferred configuration ultimately depends on the specific imaging geometry and clinical workflow.

**Table 7.** Comparison of mean (± s.d.) SSIM, RE, and TRE on the **DIR-Lab** dataset for limited-angle CBCT estimation. Five scan angle magnitudes (15°, 30°, 45°, 60°, and 90°) were evaluated for both single-view and orthogonal-view acquisitions. The single-view started at 0° (anterior–posterior), while the two orthogonal views started at 0° (anterior–posterior) and 90° (left-lateral), respectively.

DIR-Lab		15°	30°	45°	60°	90°
Single-view	SSIM↑	0.951 ±0.029	0.954 ±0.029	0.957 ±0.028	0.958 ±0.026	0.960 ±0.023
	RE/%↓	10.00 ±1.85	9.26 ±1.98	8.69 ±1.91	8.20 ±1.80	7.49 ±1.66
	TRE/mm↓	4.12 ±1.50	3.83 ±1.44	3.72 ±1.40	3.70 ±1.24	3.68 ±1.15

DIR-Lab		**15° (7.5° each)**	**30° (15° each)**	**45° (22.5° each)**	**60° (30° each)**	**90° (45° each)**

Ortho-view	SSIM↑	0.955 ±0.024	0.958 ±0.024	0.960 ±0.023	0.961 ±0.022	0.962 ±0.021
	RE/%↓	8.15 ±2.03	7.68 ±2.04	7.27 ±1.89	7.01 ±1.81	6.84 ±1.70
	TRE/mm↓	3.80 ±1.28	3.77 ±1.25	3.65 ±1.26	3.63 ±1.24	3.61 ±1.27

**Table 8.** Comparison of mean (± s.d.) SSIM, RE, and TRE under single-30°, ortho-30° (15° per view), single-90°, and ortho-90° (45° per view) limited-angle settings on the **DIR-Lab** dataset. Six starting angular offsets were evaluated: 0°, 30°, 60°, 90°, 120°, and 150° for the 30° settings, and 0°, 18°, 36°, 54°, 72°, and 90° for the 90° settings. The offset was defined relative to the anterior-posterior direction (0°) and specified the starting angle of the single view or the first orthogonal view; the second orthogonal view started 90° from the first.

DIR-Lab/30°		Off 0°	Off 30°	Off 60°	Off 90°	Off 120°	Off 150°	Max Diff
Single-view	SSIM↑ RE/%↓ TRE/mm↓	0.954 ± 0.029 9.26 ±1.98 3.83 ±1.44	0.952 ±0.026 8.92 ±1.74 3.82 ±1.36	0.949 ±0.023 9.04 ±1.61 3.85 ±1.32	0.953 ±0.022 8.63 ±1.61 3.81 ±1.26	0.956 ±0.026 8.85 ±1.80 3.74 ±1.31	0.954 ±0.029 9.10 ±2.09 3.86 ±1.29	0.007 0.63 0.12
Ortho-view (15° each)	SSIM↑ RE/%↓ TRE/mm↓	0.958 ±0.024 7.68 ±2.04 3.77 ±1.25	0.957 ±0.026 7.81 ±1.87 3.73 ±1.25	0.956 ±0.026 8.01 ±1.96 3.75 ±1.24	0.958 ±0.023 7.68 ±1.93 3.76 ±1.27	0.955 ±0.025 7.78 ±1.86 3.79 ±1.25	0.959 ±0.025 7.71 ±1.86 3.78 ±1.23	0.004 0.33 0.06

Dir-Lab/90°		Off 0°	Off 18°	Off 36°	Off 54°	Off 72°	Off 90°	Max Diff

Single-view	SSIM↑ RE/%↓ TRE/mm↓	0.960± 0.020 7.49 ±1.66 3.68 ±1.15	0.960 ±0.020 7.53 ±1.32 3.71 ±1.18	0.958 ±0.020 7.32 ±1.41 3.69 ±1.09	0.961 ±0.021 7.27 ±1.50 3.67 ±1.22	0.960 ±0.021 7.34 ±1.89 3.73 ±1.25	0.957 ±0.026 7.68 ±1.85 3.75 ±1.21	0.004 0.41 0.08
Ortho-view (45° each)	**SSIM↑** RE/%↓ TRE/mm↓	0.962 ±0.021 6.84 ±1.70 3.61 ±1.27	0.961 ±0.024 6.88 ±1.73 3.63 ±1.19	0.963 ±0.021 6.91 ±1.63 3.66 ±1.29	0.960 ±0.021 6.89 ±1.61 3.64 ±1.26	0.962 ±0.023 6.85 ±1.68 3.60 ±1.19	0.961 ±0.021 6.90 ±1.60 3.65 ±1.22	0.003 0.07 0.06

Table 9 evaluates the robustness of different methods under varying MR k-space undersampling conditions using 9- and 5-spoke per slice settings. In general, reducing the number of spokes increased the challenge of volumetric MRI estimation. Population-based methods showed sensitivity to reduced measurement information due to out-of-domain shifts, while test-time optimization-based methods generally improved performance through adaptation to new cases. DiffINR maintained comparable or superior performance under both 9- and 5-spoke settings, indicating its robustness under increasingly sparse k-space measurements.

**Table 9.** Comparison of mean (± s.d.) SSIM, RE, Dice, HD95, and COME across different methods on the **OCMR** dataset for real-time volumetric MRI estimation under 5- and 9-spoke-per-slice undersampling settings.

Number of Spokes	OCMR/Methods	SSIM↑	Re/%↓	Dice↑	HD95 (mm)↓	Come (mm)↓
5	Iterative	0.967 ±0.005	9.01 ±3.52	0.85 ±0.08	4.72 ±1.63	1.90 ±1.34
	RegNet	0.963 ±0.005	9.95 ±2.27	0.84 ±0.09	4.86 ±1.78	2.07 ±1.25
	RegNet-Ft	0.968 ±0.007	9.81 ±3.73	0.85 ±0.07	4.65 ±1.52	1.93 ±1.18
	RegNet-DVF	0.962 ±0.007	9.00 ±2.31	0.84 ±0.10	5.13 ±2.22	1.92 ±1.26
	RegNet-Gen	0.973 ±0.005	8.56 ±3.20	0.85 ±0.09	4.63 ±2.13	1.63 ±0.52
	INR	0.971 ±0.006	9.14 ±3.25	0.84 ±0.09	4.90 ±2.06	1.78 ±1.03
	cMeta-INR	0.979 ±0.006	8.22 ±2.51	0.87 ±0.08	4.08 ±1.87	1.43 ±0.97
	DiffINR	**0.982 ±0.007**	**8.17 ±3.46**	**0.88 ±0.04**	**4.07 ±1.40**	**1.41 ±1.21**

9	Iterative	0.972 ±0.007	8.92 ±3.36	0.86 ±0.09	4.59 ±1.63	1.76 ±1.13
	RegNet	0.965 ±0.006	9.71 ±2.78	0.86 ±0.08	4.77 ±1.37	1.98 ±1.25
	RegNet-Ft	0.971 ±0.006	9.63 ±3.64	0.86 ±0.06	4.58 ±1.74	1.62 ±1.12
	RegNet-DVF	0.969 ±0.008	8.48 ±3.50	0.85 ±0.11	5.07 ±1.79	1.86 ±1.14
	RegNet-Gen	0.975 ±0.006	8.33 ±3.22	0.87 ±0.09	4.55 ±1.58	1.52 ±1.30
	INR	0.977 ±0.007	8.94 ±2.05	0.85 ±0.08	4.74 ±1.62	1.75 ±1.12
	cMeta-INR	0.980 ±0.006	**7.87 ±3.64**	0.88 ±0.06	**3.85 ±1.38**	1.40 ±1.01
	DiffINR	**0.983 ±0.007**	8.05 ±3.11	**0.88 ±0.03**	4.03 ±1.37	**1.36 ±1.08**

#### 3.4.2. Test-time computational efficiency analysis

Figure 8 compares the test-time computational efficiency of different INR-based patient-specific optimization methods for both CBCT and MR imaging tasks. The reported runtimes represent per-case test-time processing and exclude the one-time offline costs of INR parameter-space construction and model training. Notably, INR from scratch required 2000 optimization epochs, whereas Hb-2D3DReg required 500 measurement-domain refinement epochs and cMeta-INR and DiffINR used only 50 refinement epochs. For the limited-angle CBCT estimation task, INR from scratch required the longest runtime of 795 s per testing case, while Hb-2D3DReg and DiffINR reduced the runtime to 197 and 23 s, respectively. For the real-time MRI estimation task, INR required 928 s, whereas cMeta-INR and DiffINR required 35 and 27 s, respectively. By leveraging learned initialization strategies, Hb-2D3DReg, cMeta-INR, and DiffINR substantially shortened the test-time adaptation process. Under the same number of refinement epochs, DiffINR required less runtime than cMeta-INR, mainly because cMeta-INR involved modulation-model optimization and a two-stage optimization procedure during test-time adaptation. These results indicated that the proposed diffusion model-generated INR initialization effectively reduced the computational burden of patient-specific optimization, supporting its potential for time-sensitive volumetric imaging applications.

**Figure 8.**
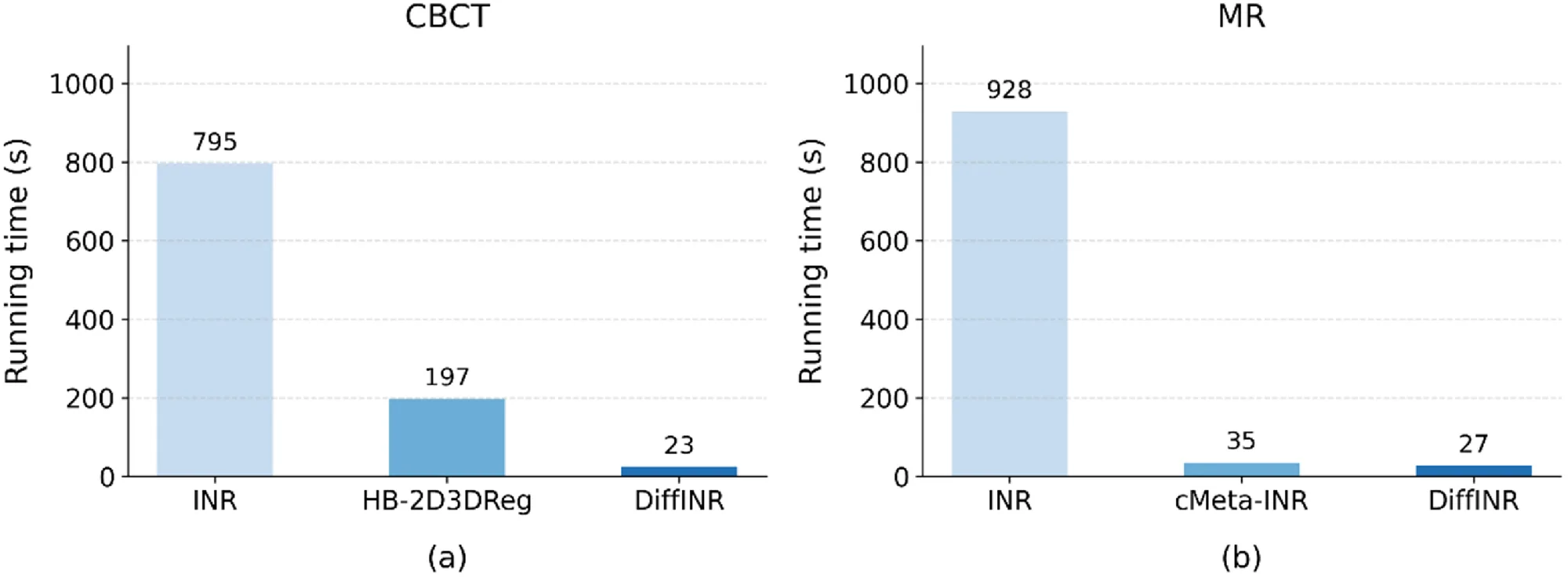
Test-time efficiency comparison of INR-based methods for (a) limited-angle CBCT estimation on the **DIR-Lab** dataset under the ortho-90° setting (45° per view) and (b) real-time volumetric MRI estimation on the **OCMR** dataset using 13 radial spokes per slice. The reported time includes all related overhead, including the inference of population-based models for INR initialization. Offline INR parameter-space construction and model training are not included.

## 4. Discussion

In this study, we proposed DiffINR, a conditional hyperdiffusion framework for deformation-driven volumetric image estimation from limited measurements. Unlike conventional patient-specific INR optimization that initializes the network randomly for each subject, DiffINR learned a conditional distribution of optimized INR parameters in the neural field weight space and generated informative initialization parameters for subsequent patient-specific refinement. By incorporating both image-domain semantic priors and measurement-domain observations, DiffINR combined population-level anatomical knowledge with patient-specific measurement constraints. To extract the image-domain semantic priors, SAM-Med3D was adopted as the foundation encoder because it provides generalizable 3D anatomical feature representations, while its frozen weights avoided additional training burden or instability on the limited task-specific datasets. This design combines population-informed initialization with patient-specific adaptation. The hyperdiffusion model provides an informative population-based prior, while subsequent measurement-domain refinement adapts the INR parameters to each individual case. The ablation results confirm the importance of learned initialization for rapid convergence and improved performance. DiffINR offers flexibility at the framework level, allowing the same overall framework to be applied to different applications, with the hyperdiffusion model generally retrained for each specific scenario. A potential future direction is to jointly train a foundation model-style hyperdiffusion model across mixed measurement scenarios, which may improve generalizability and reduce or potentially eliminate the need for scenario-specific retraining. The proposed parameter-space diffusion strategy further differs from conventional image-space or latent-space diffusion methods by generating informative INR parameters rather than an intermediate image volume. These parameters directly define a continuous implicit deformation field and provide an informative initialization for subsequent measurement-domain refinement.

The experimental results validated DiffINR on both limited-angle CBCT estimation and undersampled volumetric MRI tasks. The ablation studies confirmed the importance of the proposed components, including INR parameter space construction from fully-sampled image pairs, dual-stream conditioning, and hyperdiffusion-based initialization, followed by patient-specific refinement. Constructing a high-quality INR parameter space from fully sampled training image pairs provided a more reliable parameter distribution for diffusion learning than using parameters optimized directly from limited measurements, as limited measurements may lead to ambiguous or suboptimal DVF solutions, which can degrade the learned parameter prior. The dual-stream conditioning design, on the other hand, supported the denoising network with complementary information: image-domain priors provided semantic contexts, while measurement-domain features preserved patient-specific acquisition information. Moreover, the hyperdiffusion-generated parameters provided an informative initialization through population-level priors, and additional patient-specific refinement further enforced data consistency with the acquired measurements by adapting the INR to each individual case. In the comparison studies, DiffINR achieved superior or comparable performance to the other evaluated methods across simulated CBCT datasets, real CBCT projection data, and a cardiac MRI dataset. Visual comparisons showed that DiffINR reduced residual errors in difference maps and better recovered local anatomical details compared with population-based, iterative, and INR-based baselines. Robustness evaluations under different scan-angle magnitudes, angular offsets, and k-space undersampling levels further indicated that DiffINR maintained stable and robust performance under varying limited-sample conditions. Additional experiments using alternative reconstruction methods for the approximate target volume showed highly consistent final performance, indicating that DiffINR is relatively robust to the reconstruction approach used for image-domain conditioning (supplementary figure 1 and supplementary tables 1 and 2). Moreover, the test-time efficiency analysis showed that DiffINR reduced the optimization time compared with INR from scratch and other test-time refinement-based methods, demonstrating its ability to enable rapid patient-specific volumetric image estimation. The CBCT and MRI studies demonstrate the applicability of the general DiffINR workflow across distinct imaging tasks. In both studies, fully sampled source-target volume pairs were used for offline training, while a fully sampled source volume and limited target measurements were used at test time. The same workflow can be extended to other imaging modalities when the corresponding data are available.

While the proposed DiffINR framework demonstrated the efficacy of combining INR parameter-space diffusion with test-time INR refinement, several limitations remain and warrant further investigation. The current training and evaluation datasets were limited in size, and part of the experiments relied on simulated data. Larger and more diverse multi-institutional datasets could further help assess its generalizability across different scanners, acquisition protocols, anatomical sites, and patient populations. Another limitation is that DiffINR currently requires fully sampled training data to construct high-quality INR parameter space samples, which may not always be available in clinical practice. The multi-phase datasets in this study provided diverse source-target pairs across different motion states, while the model itself does not require temporal-phase information. For reliable performance of the population model, the training pairs should adequately represent the anatomical structures and deformation patterns expected in the target application, while subsequent case-specific adaptation can help mitigate residual gaps in data and motion representation. The current framework primarily addresses limited-data inference, while the assumption of fully sampled training targets remains a prerequisite during offline model development. Despite the relatively small training cohorts, DiffINR showed consistent performance under the evaluated settings, suggesting that the current dataset sizes were suitable for model initialization in this study. For future applications, the appropriate training-set size may be evaluated on a task-specific basis. The use of datasets acquired from different clinical centers for training and testing in the CBCT study further supports the robustness of the population-informed initialization through a hyperdiffusion model, while patient-specific test-time refinement provides additional adaptation to individual cases. Future work could explore parameter space construction and optimization strategies directly from limited measurements to reduce the dependency on fully sampled training data. Finally, although the test-time runtime was substantially reduced, the overall framework still involved multiple components, including feature encoders, conditional diffusion, DDIM sampling, and INR refinement. Simplifying the denoising network, accelerating parameter sampling, and optimizing the refinement strategy could enhance deployment efficiency and facilitate real-time clinical applications.

## 5. Conclusions

In this study, we developed DiffINR, a conditional neural-field diffusion framework that generates patient-specific INR initialization parameters in the neural field weight space for volumetric imaging from limited measurements. By combining conditional diffusion learning with subsequent patient-specific refinement, DiffINR achieved accurate and efficient volumetric image estimation. Experimental results demonstrated that DiffINR improved accuracy, robustness, and optimization efficiency, supporting its potential for rapid volumetric imaging in image-guided and adaptive radiotherapy workflows.

## Data Availability

The TCIA, Creatis, DIR-Lab, and the SPARE CBCT projection datasets used in this study are publicly available from the following sources: TCIA (www.cancerimagingarchive.net), Creatis (https://github.com/open-vv/popi-model/blob/master/dir_validation_data.md), DIR-Lab (https://med.emory.edu/departments/radiation-oncology/research-laboratories/deformable-image-registration/downloads-and-reference-data/4dct.html), and SPARE (https://ses.library.usyd.edu.au/handle/2123/34378). The OCMR cardiac dataset can be accessed at www.ocmr.info/download/. The data that support the findings of this study are available upon reasonable request from the authors. The in-house dataset is not publicly available because it contains potentially sensitive patient information and is subject to institutional restrictions. Access may be granted on a case-by-case basis upon reasonable request to the corresponding authors and approval by the institution. DiffINR supplementary data available at https://doi.org/10.1088/1361-6560/aeaaa0/data1.
